# Country‐Specific Environmental Risks of Fragrance Encapsulates Used in Laundry Care Products

**DOI:** 10.1002/etc.5168

**Published:** 2021-09-02

**Authors:** Yaping Cai, Jianming Lin, Sylvia Gimeno, Frédéric Begnaud, Bernd Nowack

**Affiliations:** ^1^ Technology and Society Laboratory, Swiss Federal Laboratories for Materials Science and Technology, St. Gallen Switzerland; ^2^ Firmenich Princeton New Jersey USA; ^3^ Firmenich Belgium, Louvain‐La‐Neuve Belgium; ^4^ Corporate R&D Division, Firmenich, Satigny Switzerland

**Keywords:** Ecological risk assessment, Environmental toxicology, Hazard/risk assessment, Microplastics

## Abstract

Fragrance encapsulates (FEs) are designed to deliver fragrance components, notably in laundry care products. They are made of thermoset polymeric shells surrounding the fragrance content. These materials enter the environment mainly during laundry washing, but little is known about their distribution in and impact on the environment. The aim of the present study was to estimate the environmental concentrations of FE shells in freshwater, sediment, and soil compartments for 34 selected countries and to compare them with ecotoxicological effects. Probabilistic material flow analysis was used to estimate worst‐case predicted environmental concentrations (PECs). The lowest freshwater PEC was predicted for Finland (0.00011 µg/L) and the highest for Belgium (0.13 µg/L). Accumulation of FE shells between 2010 and 2019 was considered for sediments and sludge‐treated soils. The PECs in sediments ranged from 3.0 µg/kg (Finland) to 3400 µg/kg (Belgium). For sludge‐treated soil, the concentration was estimated to be between 0 (Malta and Switzerland) and 3600 µg/kg (Vietnam). Ecotoxicological tests showed no effects for FE shells at any tested concentration (up to 2700 µg/L freshwater, 5400 µg/kg sediment, and 9100 µg/kg soil), thus not allowing derivation of a predicted‐no‐effect concentration (PNEC). Therefore, to characterize the environmental risks, the PEC values were compared with highest‐observed‐no‐effect concentrations (HONECs) derived from ecotoxicological tests. The PEC/HONEC ratios were 9.3 × 10^–6^, 0.13, and 0.04 for surface waters, sediments, and sludge‐treated soils, respectively, which are much below 1, suggesting no environmental risk. Because the PEC values constitute an upper boundary (no fate considered) and the HONEC values represent a lower boundary (actual PNEC values based on NOECs will be higher), the current risk estimation can be considered a precautionary worst‐case assessment. *Environ Toxicol Chem* 2022;41:905–916. © 2021 The Authors. *Environmental Toxicology and Chemistry* published by Wiley Periodicals LLC on behalf of SETAC.

## INTRODUCTION

Microplastics are a class of pollutants, defined as plastic particles with a size between 0.1 µm and 5 mm or plastic fibers possessing a length between 0.3 µm and 5 mm, with a length to diameter ratio >3 (European Chemicals Agency, 2020a). These materials have drawn a tremendous amount of attention in the last few years (Klingelhöfer et al., 2020). Depending on their origin, microplastics can be categorized into primary and secondary microplastics (European Chemicals Agency, 2020a; Joint Group of Experts on the Scientific Aspects of Marine Environmental Protection, 2015). Primary microplastics are defined as those which are intentionally manufactured into particles with a size of 5 mm or less, such as scrubbers, microbeads, and plastic powders (Amec Foster Wheeler Environment & Infrastructure, 2017; Joint Group of Experts on the Scientific Aspects of Marine Environmental Protection, 2015). However, they account for only a small part (<10%) of the microplastics found in the environment (European Chemicals Agency, 2020a). Secondary microplastics are the result of the breakdown of larger items, such tire abrasion, fibers from textiles, and fragments from weathering processes of plastic packaging products (Joint Group of Experts on the Scientific Aspects of Marine Environmental Protection, 2015), which are the major form of microplastics found in the environment (European Chemicals Agency, 2020a).

The use of fragrances has endured throughout the centuries, and various benefits of fragrance have long been recognized (Herz, 2016; Sowndhararajan & Kim, 2016). Fragrances are widely used in consumer goods such as laundry, home care, and body care products. Because many of the fragrance components are volatile and some prone to degradation (León et al., 2017; Zhao et al., 2019), fragrance encapsulates (FEs) were designed to provide long‐lasting experience, notably in laundry care products. They are mainly used in liquid fabric softeners, with additional benefits of preventing fragrance components from physical and chemical losses such as evaporation and abiotic degradation processes (León et al., 2017; Zhao et al., 2019). They are spheres with a typical diameter between 10 and 50 µm and a wall thickness <1 µm (Laroche & Gonzalez, 2018) containing the fragrance components. The shells are often made of cross‐linked thermoset melamine‐formaldehyde polymer and can also consist of other cross‐linked thermoset polymers such as polyurethane, polyurea, and polyamide, but to a lesser extent (Gasparini et al., 2020). Diverse chemistries are employed in the preparation of polymeric FEs involving phase separation polymerization, interfacial polymerization, or both (Jacquemond et al., 2009; León et al., 2017; Paret et al., 2019). Most of the microplastics found in the environment are thermoplastic polymers (de Haan et al., 2019). Although thermoset polymers and thermoplastic polymers have different properties, the European Chemicals Agency (2020b) considers FE shells as intentionally added microplastics. Fragrance encapsulates are manufactured mainly by fragrance and consumer goods companies, and the annual release of FE shells was estimated to be 200 t (130–275 t) in Europe (European Chemicals Agency, 2020b). After the use of FEs in laundry care products, they will most likely enter into the environment with the wash water and after passage through a sewage‐treatment plant. It is therefore crucial to understand the environmental fate and effects of these materials.

Considering the data available for microplastics in wastewater‐treatment plants, only approximately 10% of FE shells are expected to be released into surface waters because the major fraction of microplastics (~90%) is removed by sorption to the sludge during wastewater treatment (Lassen et al., 2015; Bläsing & Amelung, 2018). Because sludge is commonly used as fertilizer and >50% of the sludge is applied on agricultural soil in Europe and North America (Nizzetto et al., 2016), a large fraction of FE shells is likely to end up in soils. So far, there is no study available identifying the occurrence of FEs in environmental samples. Although the release of FE shells is insignificant when compared to the total volume of microplastics (42 000 t/year) released in Europe (European Chemicals Agency, 2020c), representing <0.03% of the microplastics, the environmental fate of these materials should be addressed to provide guidance for the development and selection of ecofriendly FEs.

Because microplastic measurements are often spatially inhomogeneous and the information for certain environmental compartments (e.g., soil) is limited, modeling can be a valuable substitute to estimate the exposure level of microplastics in the environment. A recent study using probabilistic material flow analysis (PMFA) quantified the mass flows of macro‐ and microplastics released from a large variety of products to the environment (Kawecki & Nowack, 2019). The emission of macroplastics to freshwater and soil in Switzerland was estimated to be 109 and 4400 t/year, respectively, which was approximately 7 times higher than the emission of both primary and secondary microplastics (freshwater, 15 t/year; soil, 600 t/year; Kawecki & Nowack, 2019). Construction pipes (137 t/year), agricultural films (114 t/year), and postconsumer processes (e.g., littering; 89 t/year) ranked as the 3 largest sources of microplastics in soil, whereas for microplastics in freshwater, littering (3.6 t/year), clothing fibers (3.1 t/year), and microbeads in personal care products (2.6 t/year) were the biggest contributors (Kawecki & Nowack, 2019). It should be noted that in the meantime microbeads in personal care products have been banned in several countries and will not be a source of microplastic anymore (European Chemicals Agency, 2019).

Ecotoxicity of microplastics has been investigated for both aquatic and terrestrial organisms (Joint Group of Experts on the Scientific Aspects of Marine Environmental Protection, 2015; Anbumani & Kakkar, 2018; Wang et al., 2020; Xu et al., 2020). The ecotoxicological effects of plastic debris range from no observed effects to effects at the cellular and organism levels (Bucci et al., 2020). The greatest concern regarding microplastic pollution is the risk of ingestion (von Moos et al., 2012; Farrell & Nelson, 2013; Setala et al., 2014; Peng et al., 2017), which can result in several negative effects. There is evidence that ingestion of microplastic fibers by terrestrial snails can result in decreased food intake (Song et al., 2019), whereas the uptake of microbeads by mussels can lead to inflammation (von Moos et al., 2012). Other detrimental effects such as mucosal damage, longer egestion times, and reduced growth have also been reported (Au et al., 2015; Ziajahromi et al., 2017; Qiao et al., 2019). However, some experiments applied unrealistically high concentrations, which raises questions about their environmental relevance (Lenz et al., 2016).

Currently, only a very limited number of risk assessments of microplastics have been published in the peer‐reviewed literature (Burns & Boxall, 2018; Everaert et al., 2018; Adam et al., 2021). The predicted‐no‐effect concentration (PNEC) for freshwater was calculated based on species sensitivity distributions, which was 4.2 × 10^–2^ µg/L (Adam et al., 2019). This PNEC was obtained by considering all types of microplastic spheres, fragments, and fibers tested in toxicological experiments with a size mainly between 0.1 and 1000 µm. Risk assessment showed that only a very small percentage (0.12%) of the probability distribution calculated for the global risk characterization ratio was >1 (Adam et al., 2019). However, the study by Adam et al., (2019) provides a generic risk assessment for all types of microplastics without distinguishing between polymer types. The environmental risks posed by FEs made of thermoset polymers have so far not been quantified.

The aim of the present study was to estimate the environmental exposure levels of FE shells in freshwaters, sediments, and soils for 34 selected countries and to compare the environmental concentrations with ecotoxicological effects. A PMFA model (Gottschalk et al., 2010) was applied to estimate the flows of FE shells to the environment and to obtain the predicted environmental concentrations (PECs). Ecotoxicological tests with FE shells were conducted to measure potential effects on freshwater, sediment, and soil organisms. Finally, the environmental risk was characterized by comparing the PEC values to ecotoxicological threshold values.

## METHODS

### Material flow model description

**Figure 1:**
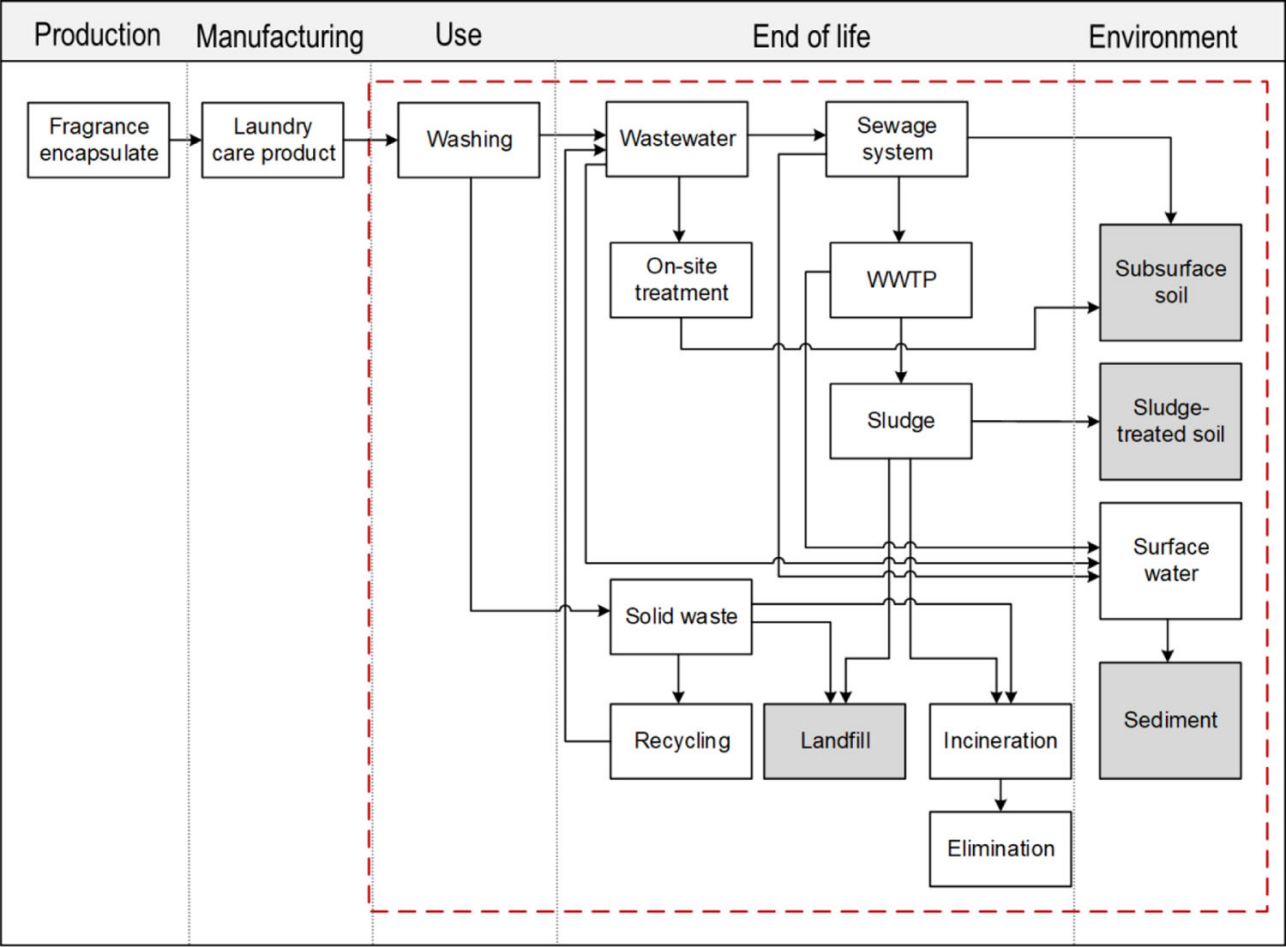
Definition of the system. The system boundary is outlined by the red dashed line. Sinks are labeled as gray boxes. WWTP = wastewater‐treatment plant.

A PMFA model was programmed on the platform R (R Development Core Team, 2013), based on the basic PMFA algorithm by Gottschalk et al. (2010). The model was developed to assess the mass flows of anthropogenic materials to the environment through their complete life cycle, from production, manufacturing, and use to the end‐of‐life management and the release to the environment. The model was adapted and parameterized for FE shells (Figure 1). The release during FE production and laundry care product manufacturing was estimated to be <0.1% of the total production and was not considered in the present study. The assessment was performed at a national scale for 34 selected countries including the 27 countries of the European Union, the United Kingdom, Switzerland, Norway, the United States, Mexico, Japan, and Vietnam, where FEs are used in laundry care products. These countries were selected because of their specific habits representing various continents (surrogates for consumer habit, infrastructure, and release). In some countries a large portion of gray wastewater is directly discharged to the environment. Four waste‐treatment processes are generally present: wastewater‐treatment plants (WWTPs), incineration, landfilling, and recycling. As organic material, FE shells ultimately decompose to water and carbon dioxide at a high temperature; therefore, FEs flowing into incineration plants are eliminated, which also applies to FE shells contained in sludge that is incinerated. The landfill was considered a final sink for the scope of our model. The presence of microplastics in leachates of municipal solid waste landfill has been confirmed in China (0.42–24.58 items/L) and the Nordic countries (0–0.17 µg/L for treated leachate; Praagh et al., 2018; He et al., 2019), but the correlation of the total amount of microplastics landfilled compared to the amount leached is unknown. Because recycling solely applies to the plastic containers where the laundry care products are contained, the flow of FE shells out of plastic recycling was considered as wastewater based on the model by Rajkovic et al. (2020). A small fraction of laundry care products (including FE shells) remains in the containers and is removed in washing steps during recycling. Three environmental compartments were included in the model: surface water, sediment, and soil. For soil, only the concentration in sludge‐treated soil was considered because sewage sludge is widely used in many countries as organic fertilizer (Nizzetto et al., 2016). The use of gray water for soil irrigation was not considered because of lack of data.

### Input parameters

#### Production, manufacturing, and consumption volume

Fragrance encapsulates are a technology to protect fragrance components from evaporation and degradation (Ji et al., 2021) and are reported to be mainly used in laundry care products (European Chemicals Agency, 2019). According to sales data, >95% of FEs are used in laundry detergents and fabric softeners. Of the total European Union market, approximately 10 to 20% of laundry detergents and approximately 60% of fabric softeners contain FEs, whereas <1% of deodorants and other cosmetic, personal care, and cleaning products contain FEs (Laroche & Gonzalez, 2018). In the model, we therefore assumed that all of the FEs were used in laundry care products.

The total mass of FE shells released in Europe for the year 2019 was calculated by the European Chemicals Agency (2020b) according to the information reported by industry. The mass released in each European country was estimated from the total mass released in Europe and the relative retail volumes of fabric softeners in European countries obtained from Euromonitor International (2020) because fabric softener is the main application for FEs. For the other 4 countries outside of Europe (USA, Mexico, Vietnam, and Japan), the mass of FE shells released was calculated based on the country‐specific release mass of one of the major manufacturers and the estimated market share of the manufacturer (Supplemental Data, Table S1). A triangular distribution was introduced into the model to address uncertainties of the release volume. The mode, minimal, and maximum values of the distribution were defined as the mean and the lower and upper boundaries provided by the original data set, respectively. For the 4 non‐European countries where only the mean value was available, an uncertainty of 50% was assumed as the width of the distribution.

To assess the accumulation of FE shells over the years in sediments and soils, the historic releases of FE shells were estimated. The releases from 2010 to 2019 were scaled from either the global (for the 27 European Union countries, the United Kingdom, Norway, and Switzerland) or the country‐specific (for the United States, Mexico, Japan, and Vietnam) FE shell consumption trends shown in Supplemental Data, Table S2. These trends were calculated based on technical data, sales, and estimated market shares of one of the major manufacturers. Because the market for FEs was only well established after the year 2015, an additional uncertainty of 5%/year was applied to all countries for the years starting from 2015 and going backward. The uncertainty increased with the distance of the year from 2015 by 5% (i.e., 5% for 2015, 10% for 2014).

#### Transfer coefficients

Transfer coefficients describe the distribution of product flows at all life‐cycle stages, depending on the product application. Softeners containing FEs are used during washing, and 95% of FEs in softeners were assumed to flow to the wastewater. The other 5% of FEs were assumed to remain in the package and to be discarded as solid waste (Rajkovic et al., 2020). The WWTP removal efficiency of FE shells was estimated based on the removal efficiency of microplastics. The selection of studies was based on 2 high‐quality reviews published recently (Cristaldi et al., 2020; Iyare et al., 2020), where the removal efficiencies from 81 WWTPs around the world were listed based on 26 publications (Supplemental Data, Table S3). The removal efficiencies range from 35 to 99%, with an average of 90%. A distribution was constructed by randomly sampling from the 81 collected values. The other transfer coefficients at the end‐of‐life stage were obtained either from the Eurostat, Organisation for Economic Co‐operation and Development (OECD), and European Environment Agency databases for European countries or from the literature for the 4 non‐European countries (United States, Mexico, Japan, and Vietnam; Supplemental Data, Figures S1–S3, Tables S4–S9). Data from the latest years were always preferred in the data collection. Similar to the consumption volume, a distribution was introduced to reflect the uncertainties. A triangular distribution was applied for other transfer coefficients, where the mode of the distribution was defined by the value collected from databases or the literature, whereas an uncertainty of 50% was defined as the width of the distribution.

#### Volume of environmental compartments

To predict the environmental concentration in surface water, sludge‐treated soil, and sediment, volumes of each compartment were collected for all countries considered in the model. The area of the surface water was obtained by averaging the inland water area for each country from the “land use” and “land cover” databases from the Food and Agriculture Organization, (2019a, 2019b; Supplemental Data, Table S10). The sediment area was assumed to be equal to the area of surface water. The area of sludge‐treated soil was obtained by dividing the sludge volume in each country (Supplemental Data, Table S11) with the average sludge application rate (5000 kg/ha; European Chemicals Agency, 2016). To calculate the volumes of the environmental compartments, the depth of water, sediment, and soil was taken as suggested by the Registration, Evaluation, Authorisation and Restriction of Chemicals guidance (European Chemicals Agency, 2016). For freshwaters, a residence time of water (40 days) was applied as suggested by the European Chemicals Agency (2016).

Worst‐case scenarios were used to estimate PEC values excluding any possible fate processes in the environment: 1) for freshwater, complete suspension of the FE shells was considered (conservative scenario without any removal processes); 2) for sediment, complete sedimentation of FE shells from surface water was considered; and 3) for soil, no biodegradation or other degradation reactions were considered, and only accumulation of FE shells was modeled. All compartments were assumed to be homogenous and well mixed. The concentration for freshwater was predicted from a static model that only considered the release in the year 2019. For sediments and soils, a dynamic approach was used to estimate the concentration considering the accumulation of FE shells from previous years (2010–2019). This dynamic modeling was based on the approach developed by Gottschalk et al. (2009). To obtain a probabilistic distribution of PECs, 100 000 iterations of models were run.

### Hazard assessment

Ecotoxicological experiments were carried out at Fraunhofer Institute for Molecular Biology and Applied Ecology (Germany), and the detailed information can be provided on request. The tests were conducted for 10 species in freshwater, sediment, and soil using 2 types of FE suspensions. Typical melamine‐formaldehyde fragrance‐delivery systems used in laundry care products were used to prepare the test material. A protocol for preparing such an FE suspension at the lab scale has been reported by Gasparini et al. (2020).

#### Test material

Fragrance encapsulations utilized in consumer products are sold as a slurry that contains the encapsulations suspended in water plus additives (<5%) such as colloidal stabilizer, deposition aid materials, and preservatives. Encapsulations are thermoset polymers forming a shell of a thickness generally well below 1 µm surrounding a liquid fragrance droplet (total average diameter 10–25 µm). The shells are designed so that the contained fragrance is released by the action of friction or pressure, which will rupture the shells. Because the test material of interest is one composing the polymeric shells, a protocol was developed to remove the fragrances and the additives from the capsules (Gasparini et al., 2020). The protocol consisted of several steps of drying, grinding, and extraction by organic solvents or water. The resulting purified and ground test material was light yellow fine powder of roughly spherical particles. The diameter of the particles was approximately 1 µm, and the density of the powder was estimated by ultracentrifugation to be 1.3 to 1.4 mg/L. The test materials were therefore a suspension of FE shells in water with (commercial) or without (purified) additives.

#### Ecotoxicological tests

Rangefinder tests were first performed with the commercial suspension of FE shells. The ecotoxicological tests were conducted following OECD tests 201 (algal growth inhibition test with *Raphidocelis subcapitata*), 202 (*Daphnia magna* immobilization), 236 (zebrafish embryo, *Danio rerio*), 209 (sediment test with the sediment dweller *Lumbriculus variegatus*), 208 (plant growth inhibition with 4 species, i.e., oat [*Avena sativa*], corn [*Zea mays*], bean [*Phaseolus aureus*], and turnip [*Brassica rapa*]), and 217 (soil microbial carbon transformation), and 222 (worm reproduction test with the earthworm *Eisenia andrei*) as well as ISO 15685 (soil microbial nitrogen transformation). The soil used was Hagen AM 2013 (loamy sand [US, DIN], 73% sand, 22% silt, 5% clay, and 1.1% C_org_, 1.2 g N/kg). The soils were preloaded with test material at a concentration of 0.5 to 5.35 mg FE shells/kg soil. When any adverse effects were observed, the test was repeated with a broader range of concentrations and with a purified suspension of FE shells, thus without additives to rule out their potential effect. Both suspensions had a concentration of 5.35% FE shells. An overview of the tests with test duration and endpoint is given in Table 1.

**Table 1:** Overview of ecotoxicological tests

Compartment	Guideline	FE shell suspension	Test species	Endpoint	Duration	Concentration range (mg FE shells/L water or/kg soil or sediment)
Freshwater	OECD 201	Commercial	*Raphidocelis subcapitata*	Growth	72 h	0.00535–0.0535
Freshwater	OECD 201	Purified	*Raphidocelis subcapitata*	Growth	72 h	0.0535–2.67
Freshwater	OECD 202	Commercial	*Daphnia magna*	Immobilization	72 h	0.00535–0.0535
Freshwater	OECD 236	Commercial	*Danio rerio*	Survival or growth	96 h	0.00535–0.0535
Sediment	OECD 225	Commercial	*Lumbriculus variegatus*	Mortality and weight	28 d	0.535–5.35
Soil	OECD 217	Commercial	Soil microorganisms	Carbon transformation	28 d	0.535–5.35
Soil	ISO 15685	Commercial	Soil microorganisms	Nitrogen transformation	28 d	0.535–5.35
Soil	OECD 208	Commercial	*Avena sativa*	Growth	14 d	0.535–5.35
Soil	OECD 208	Purified	*Avena sativa*	Emergence, survival, and growth	14 d	1.09–9.1
Soil	OECD 208	Commercial	*Zea mays*	Emergence, postemergence survival, growth	14 d	0.535–5.35
Soil	OECD 208	Commercial	*Phaseolus aureus*	Shoot fresh wt	14 d	0.535–5.35
Soil	OECD 208	Purified	*Phaseolus aureus*	Emergence rate, postemergence survival, and biomass	14 d	1.09–9.1
Soil	OECD 208	Commercial	*Brassica rapa*	Emergence rate, postemergence survival, and biomass	14 d	0.535–5.35
Soil	OECD 222	Commercial	*Eisenia andrei*	Reproduction	56 d	0.535–5.35
Soil	OECD 222	Purified	*Eisenia andrei*	Reproduction, survival, and weight	56 d	1.09–9.1

FE = fragrance encapsulate; OECD = Organisation for Economic Co‐operation and Development.

## RESULTS

### Exposure assessment

#### Consumption volume

**Figure 2:**
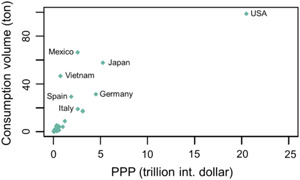
Consumption volume of fragrance encapsulates (tons of shells) versus the purchasing power parity (PPP) for each country in the year 2019. The PPP was collected from World Bank (2019).

The present study provides the first environmental exposure and hazard assessment of FE shells. It concerns a material that according to the European Chemicals Agency's definition has to be considered as a primary microplastic. Fragrance encapsulates are used worldwide, and the consumption of FEs generally increases with purchasing power parity (PPP; Figure 2). The mean consumption volume of FE shells in the year 2019 for European countries range from 0.14 t for Malta to 31 t for Germany with a total volume of 165 t. Of the 4 non‐European countries, the highest annual release was found for the United States (99 t), followed by Mexico (66 t) and Vietnam (46 t). It is interesting to note that Mexico and Vietnam consume a considerable amount of FEs, although both countries have a much smaller PPP compared with many countries. The consumption of liquid fabric softeners, which is one of the main uses of FEs, is strongly influenced by the laundry care habits of different cultures.

The historic consumption of FEs between 2010 and 2019 was estimated using the consumption volume of 2019 (Supplemental Data, Table S1) and the sales trends (Supplemental Data, Table S2). For European countries, the historic release volumes were estimated from the global sales trend of one major manufacturer, resulting in a total release of 1249 t over the 10 year. For the 4 non‐European countries, the historic consumption was estimated from country‐specific sales trends, and the cumulative consumption volume was found to range from 249 t for Japan and 693 t for the United States. Because there is no information indicating if all the products were used up within a year, the assumption was made to equate consumption and release.

#### Fragrance encapsulate mass flows in 2019

**Figure 3:**
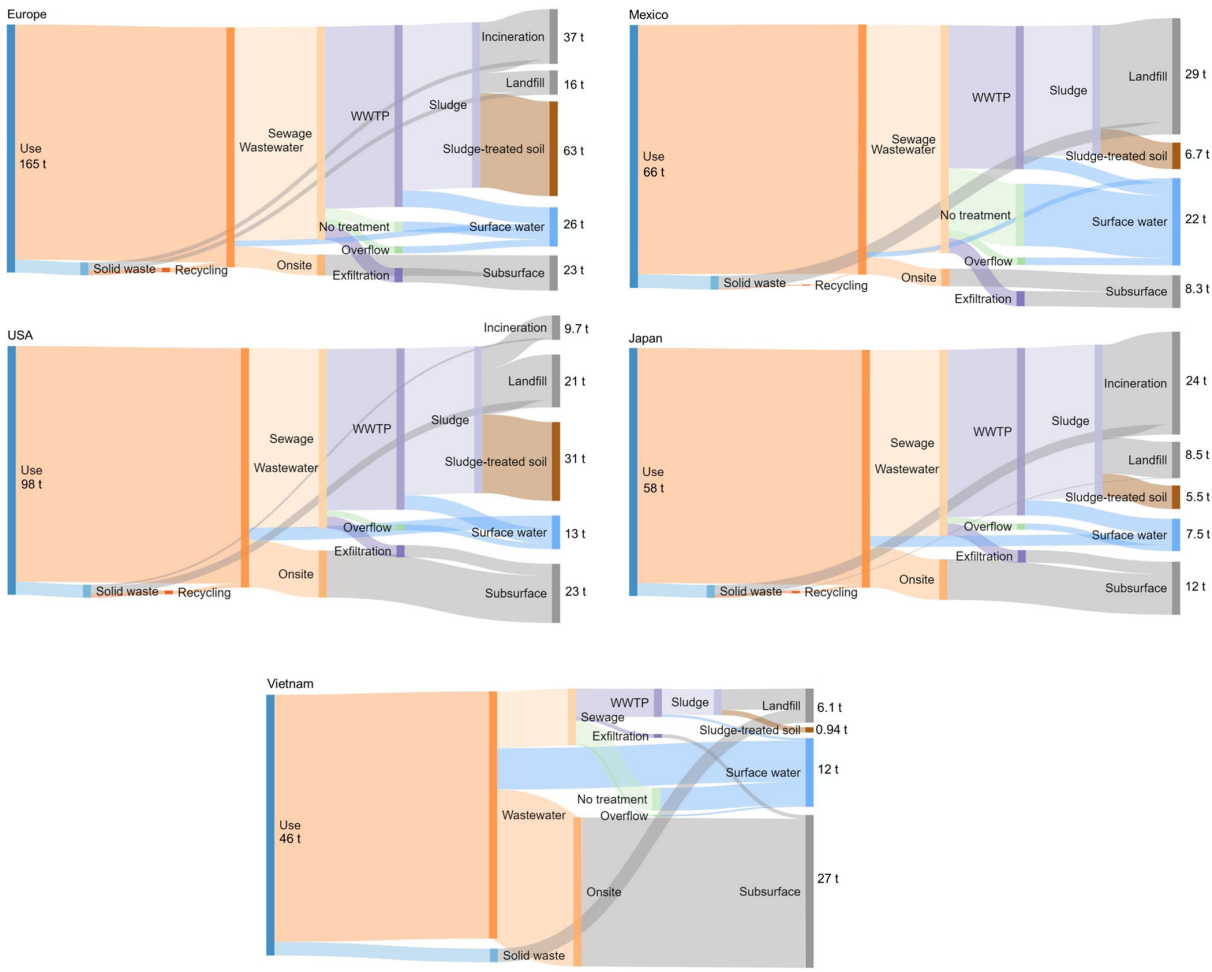
Flow diagrams of fragrance encapsulate shells in Europe (average), the United States, Mexico, Japan, and Vietnam in the year 2019. Units are tons per year. WWTP = wastewater‐treatment plant.

Fragrance encapsulates flowed into the system through “production” and were distributed to technical and environmental compartments depending on transfer coefficients. The mode, mean, Q5, and Q95 of the distribution were extracted from the probability distributions of each mass flow. Figure 3 shows simplified flow diagrams which give an overview of the differences in the flows from consumption to the receiving compartments for the different regions/countries. The relative importance of flows to different compartments varied highly among regions (Supplemental Data, Table S12). For Europe and the United States, the dominant flow was to sludge‐treated soil, which accounted for 38% (63 t) and 32% (31 t) of the total flows, respectively, whereas for Japan, Mexico, and Vietnam, the mass flowing to the sludge‐treated soil was <10%. On the other hand, the flow to surface water accounted for 10 to 20% of the total mass released for most of the selected countries, except for Mexico, where >30% of the capsules ended up in surface water. For Mexico and Vietnam, the flow to incineration plants was zero.

#### PEC

**Figure 4:**
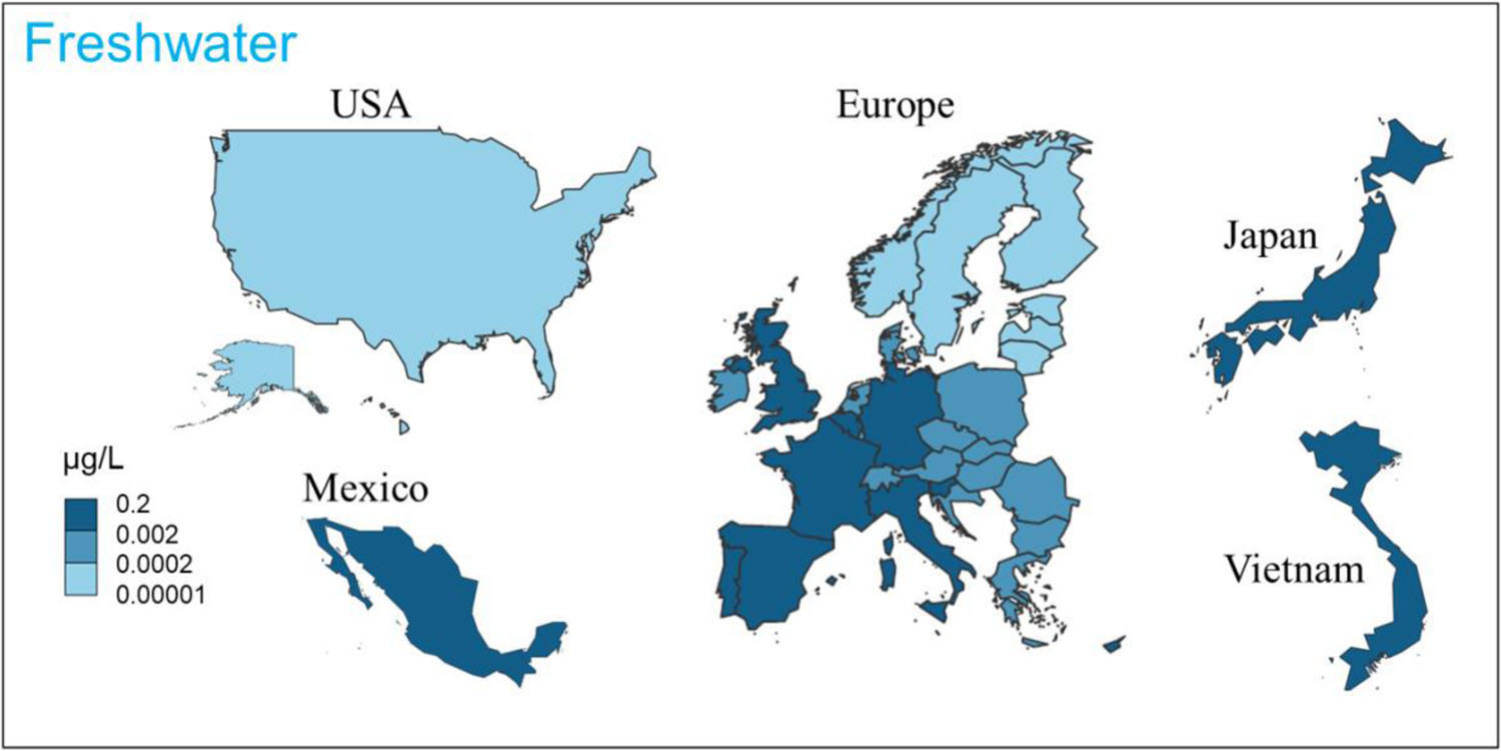
Predicted environmental concentration values for fragrance encapsulate shells in freshwater for 34 countries based on the flow of 2019.

The PEC values of the FE shells were estimated by dividing the aggregated mass flows to the environmental compartments by the mass or volume of the corresponding compartment (Sun et al., 2014), representing concentrations in well‐mixed systems. The PEC values were estimated for the 3 environmental compartments freshwater, sediment, and sludge‐treated soil. For freshwater, country‐specific PECs were calculated for the year 2019, as shown in Figure 4 and Supplemental Data, Table S13. The highest concentration was found for Belgium (0.13 µg/L), followed by Malta (0.12 µg/L), and the lowest value was found for Finland (0.00011 µg/L). The European countries had an average concentration of 0.025 µg/L. For the 4 countries outside of Europe, a relatively low concentration was found for the United States (0.0010 µg/L), whereas for Mexico, Japan, and Vietnam, the values were between 0.026 and 0.047 µg/L.

**Figure 5:**
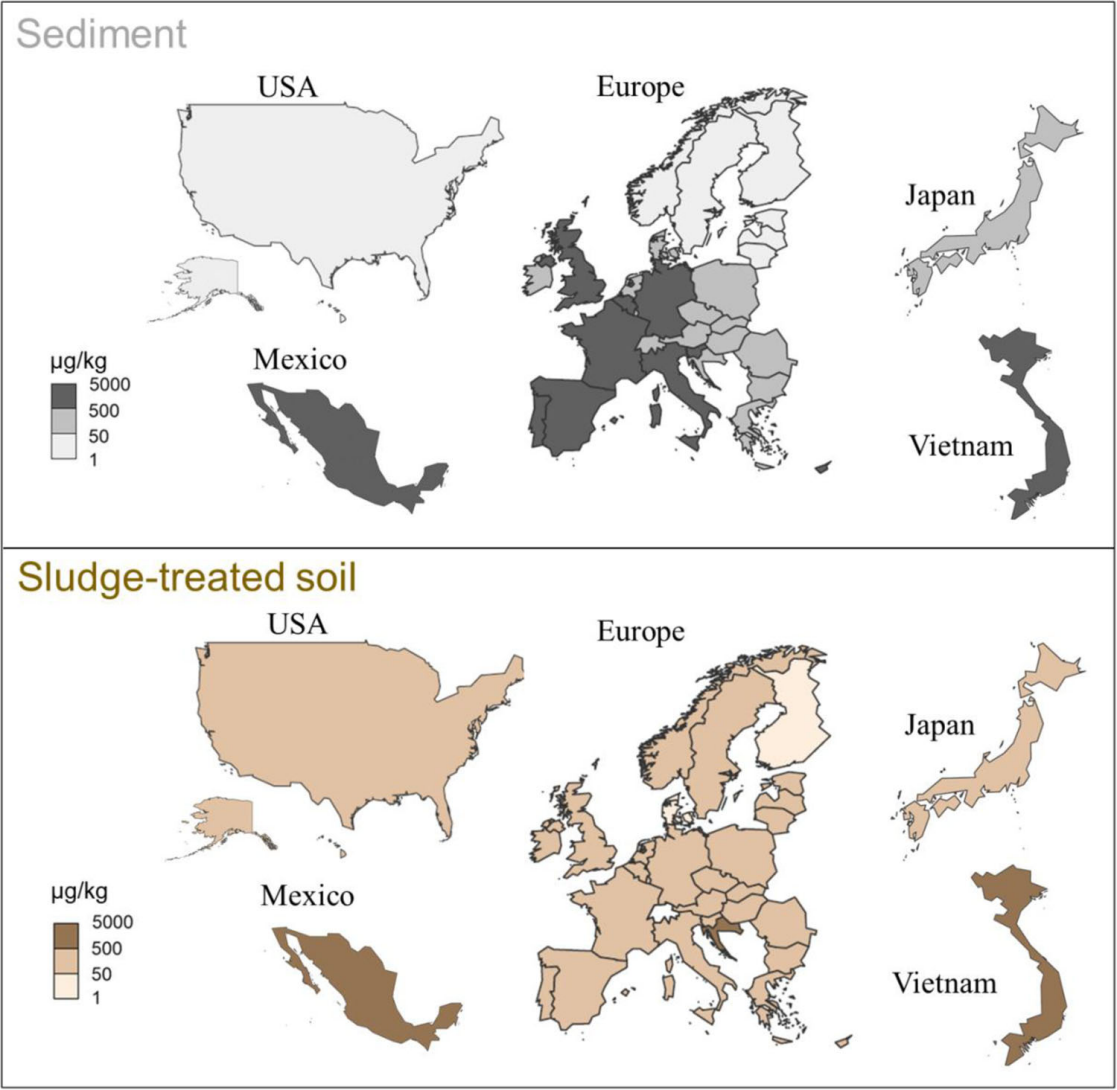
Predicted environmental concentration values for fragrance encapsulate shells in sediment and sludge‐treated soil considering accumulation for 34 countries over the years 2010 to 2019.

Accumulation of FE shells was considered for sediment and sludge‐treated soil. The PECs were calculated by dividing the sum of the mass flow over the years from 2010 to 2019 by the mass of sediment and sludge‐treated soil compartments for each country. The PECs are shown in Figure 5, Supplemental Data, Tables S14 and S15. Similar to surface water, the highest concentrations for sediment were found for Belgium (3400 µg/kg) and Malta (3300 µg/kg), whereas the lowest value was found for Finland (3.0 µg/kg). A mean value of 650 µg/kg was calculated for the 30 European countries, whereas for the countries outside Europe the concentration ranged from 29 µg/kg (United States) to 1500 µg/kg (Vietnam).

For sludge‐treated soil, countries including Malta and Switzerland had zero concentration because the sludge is not applied on agricultural soil. The highest concentration in sludge‐treated soil worldwide was found for Vietnam (3600 µg/kg). The 30 European countries had an average PEC of 210 µg/kg for sludge‐treated soil. The United States had the lowest concentration (100 µg/kg) outside Europe, followed by Japan (110 µg/kg).

To explore the contributing factors that may influence the prediction of PECs, we plotted correlation figures for various parameters including population density, consumption, consumption per capita, percentage of sludge applied on soil, total sludge volume, volume of sludge applied on soil, area of surface water, sewer connection rate, WWTP treatment rate, and precipitation. The plots can be found in Supplemental Data, Figure S4. The relevant statistics are summarized in Supplemental Data, Table S16. Although some parameters demonstrated a certain degree of significance, none of the regressions had an *R* ^2^ > 0.5, suggesting only a poor correlation with single parameters. The PEC values are influenced by multiple factors at the same time. Therefore, each country has its unique situation, and it is not possible to draw a general conclusion applicable to all countries.

### *Hazard assessment: Results of* ecotoxicological tests

The results from ecotoxicological tests Investigating the polymeric material of the FEs are provided in Supplemental Data, Table S17. All tests met the validity criteria, except the plant test with oat (*A. sativa*) because of a low control seedling emergence (63.3% vs the minimum value of 70%). Thus, the test was repeated. In most of the range‐finder tests, no effect was observed even at the highest test concentrations. However, the shoot weight of the bean was reduced at the highest concentration, and worm reproduction was reduced at the medium and highest concentrations. Therefore the bean test and the worm reproduction test were also repeated with a higher number of concentrations and with FE shells without additives. In these repeat full tests, no adverse effects were observed, and remarkably plant growth was promoted for both the oat and bean at most concentrations.

The endpoints from the full tests with the purified FE shell suspensions were preferred in the hazard assessment to focus on the polymeric materials. Therefore, 5 of the endpoints from the 3 tested environmental compartments were considered to be most relevant to evaluate the hazard effects posed by FE shells (Table 2). Because no effects were observed, no‐observed‐effect concentration (NOEC) or 20% effect concentration (EC20) values cannot be provided; but the highest tested concentrations can be used as a highest‐observed‐no‐effect concentration (HONEC). In the absence of NOEC values, HONECs have been used to derive PNEC values in a precautionary worst‐case approach (Wigger et al., 2020). For freshwater and sediment, chronic HONECs were obtained from tests with algae (*R. subcapitata*, 2.67 mg/L) and a sediment worm (*L. variegatus*, 5.35 mg/kg). Three data points tested with 3 different species were collected for soil. The only chronic HONEC for the soil compartment is for the earthworm (*Eisenia andrei*) with a value of 9.1 mg/kg.

**Table 2:** Most relevant ecotoxicological endpoints of fragrance capsules for assessing their environmental risks

Environmental compartment	Species	Duration	Chronic/acute	Descriptor	Concentration
Freshwater	*Raphidocelis subcapitata*	72 h	Chronic	HONEC	2.67 mg/L
Sediment^a^	*Lumbriculus variegatus*	28 d	Chronic	HONEC	5.35 mg/kg
Soil	*Eisenia andrei*	56 d	Chronic	HONEC	9.1 mg/kg
	*Zea mays*	14 d	Acute	HONEC	9.1 mg/kg
	*Avena sativa*	14 d	Acute	HONEC	9.1 mg/kg

^a^ Experiment conducted with the commercial suspension.

HONEC = highest‐observed‐no‐effect concentration.

### Risk characterization

**Figure 6:**
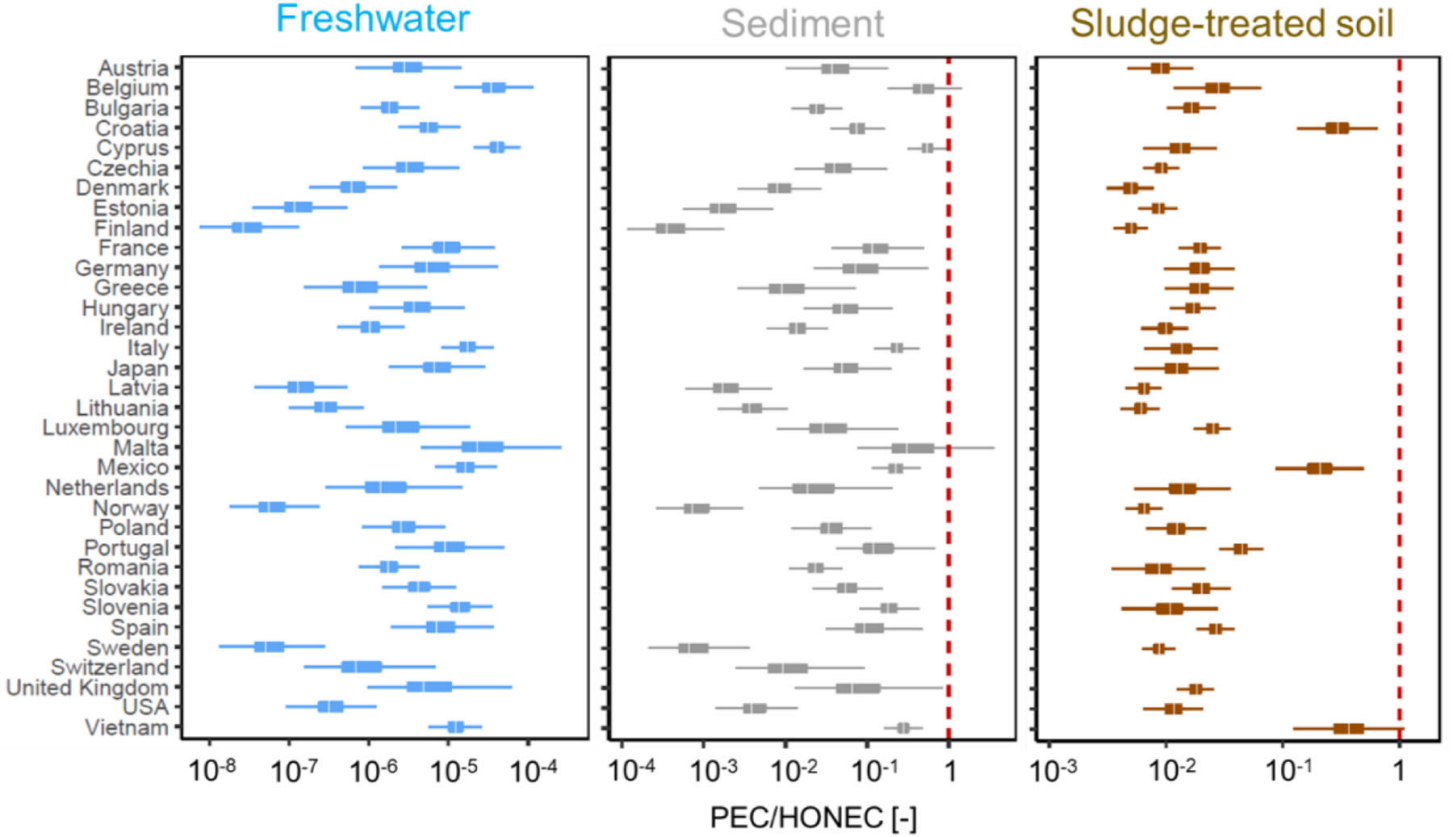
Distribution of predicted environmental concentration to highest‐observed‐no‐effect concentration ratios for 34 countries. Boxes represent 25th and 75th percentiles of the distribution, and a white line in a box indicates the median value. Whiskers represent 95% of the distribution. Red line notes that the ratio = 1. PEC = predicted environmental concentration; HONEC = highest‐observed‐no‐effect concentration.

To characterize the risks for each compartment, the distribution of the PEC values was divided by the HONECs (Figure 6; Supplemental Data, Tables S18–S20). The mean of the PEC/HONEC ratio for surface water for 34 counties was 9.3 × 10^–6^ (4.2 × 10^–8^ to 4.7 × 10^–5^), which is several orders of magnitude lower than 1, suggesting a lack of risk in the environment because of the use and release of FEs. The PEC/HONEC ratios for sediment were higher than those for freshwater, ranging from 5.6 × 10^–4^ for Finland to 0.63 for Belgium, with an average of 0.13 worldwide. Sludge‐treated soil had slightly lower PEC/HONEC ratios than sediment, with a mean value of 0.04 for 34 countries. Except for those countries that had zero soil concentration, the lowest value was found for Denmark and Finland (4.9 × 10^–3^), whereas the highest was calculated for Vietnam (0.39). For some countries, such as Belgium and Malta, there was a small percentage of PEC/HONEC ratios >1 for sediment (Belgium, 14%; Malta, 17%).

## DISCUSSION

Using material flow analysis, the flows of FEs to each environmental compartment were determined by transfer coefficients. Relatively high‐quality data were available for wastewater and sludge treatment for European countries, the United States, and Japan, which means that the data could be acquired from sources such as Eurostat or peer‐reviewed publications. However, for Mexico and Vietnam, the openly accessible information regarding wastewater management and sludge disposal was extremely scarce. Although some data could be found, it was difficult to get information on the complete system. For instance, a report covered wastewater management in Vietnam, but only the situation in urban areas was addressed (ARCOWA, 2018). The urban population for Vietnam only accounted for 38% of the total population in 2019 (World Bank, 2020), which means that information regarding the wastewater disposal for a large region of the country was missing. Therefore, assumptions had to be made that the sewage connection and the wastewater‐treatment rate in rural areas in Vietnam were zero. The chosen approach is therefore a conservative one resulting in worst‐case emissions. Moreover, the sludge is reported to be not removed regularly from septic tanks, and even if it is removed, the illegal dumping of sludge is a very common practice in Vietnam (ARCOWA, 2018). This may also result in a percentage of FEs released from illegal dumping of sludge into soil and freshwater, and this model does not consider this flow because of limited data. More information is required to obtain a more accurate prediction.

The fraction of FEs removed from wastewater was assumed to be the same as that of microplastics. The available data on the removal of microplastics during wastewater treatment cover a wide range from 25 to 5000 µm (Cristaldi et al., 2020; Iyare et al., 2020). Because the size of FEs is between 10 and 50 µm (Laroche & Gonzalez, 2018), the accuracy of the predicted removal efficiency could be improved if size‐dependent removal efficiencies were available. Despite the fact that there are many studies reporting the size of microplastics in WWTPs, not much is known about the removal efficiency for microplastics with different sizes. For instance, a study by Long et al. (2019) reported that microplastics with smaller size tended to be better removed by WWTP, whereas the opposite trend was observed by Magni et al. (2019), where the removal efficiency for microplastic with a size between 5 and 1 mm (94%) was 1.4 times higher than that for a size between 0.1 and 0.01 mm (65%). Therefore, future studies are needed to better understand how the size of microplastics influences the removal efficiency.

Although there is no other study available addressing specifically the environmental flows of FEs, efforts have been made to understand the source and release of microplastics into the environment. A recent study quantified the polymer‐specific flows of microplastic to water and soils for Switzerland (Kawecki & Nowack, 2019). Compared with the annual flows of microplastics from clothing (3.1 t/year) and personal care products (2.6 t/year) to surface waters in Switzerland, the mass of FE shells released to Swiss surface waters based on our model is only 0.16 t/year. The released FE shells correspond to approximately 5 to 6% of microplastics released from clothing or personal care products and only 1% of the total microplastic emission into surface waters (14.9 t/year), as modeled by Kawecki & Nowack (2019). If tires are also considered with a predicted release of microrubber into Swiss surface waters of 1800 t/year (Sieber et al., 2020), the contribution of FEs to the total microplastic release to the environment is marginal. Therefore, FEs make up only a very small fraction of all microplastics released into surface water. In addition, the major part of the microplastics found in the environment are expected to be formed by degradation of macroplastic (Ter Halle et al., 2016), and thus the contribution of FEs to the microplastic burden in the environment will be even smaller (<<0.009%).

Using our PMFA model, we are able to provide a first estimate of PEC values for FEs, but it has to be kept in mind that they represent a worst‐case scenario without considering any degradation in the environment. The PEC calculation for water assumes that all FE shells remain suspended in water and that for sediment that all FE shells in water had sedimented out of the water column. Both constitute, therefore, the upper boundary of environmental concentrations that could possibly be observed. In reality, not all FEs would remain suspended, and therefore the water concentration would be reduced. The PEC values assumed one well‐mixed compartment per country. Local hotspots (e.g., after wastewater input into a small river) are not captured by the model.

The estimated PEC values varied greatly among countries. It is not surprising to find that the PECs were influenced by the country‐specific wastewater management and sludge disposal. For instance, the concentration of FE shells was predicted to be zero for the soil compartments of some countries including Malta and Switzerland. That is because sludge is not allowed to be applied on soil in those counties, resulting in a zero flow of FEs to soil. On the other hand, it is interesting to note that although the amount of mass flowing to some environmental compartments was comparable in some countries, the estimated PECs can be very different. For example, the amount of FEs flowing to surface water was 0.67 and 0.71 t for Belgium and Portugal, respectively, whereas Belgium had a PEC of 0.13 µg/L for surface water, which was 3 times higher than that for Portugal (0.04 µg/L). The reason behind this difference was that the PEC calculation was influenced not only by the mass flowing to the aquatic compartment but also by the mass of the receiving water systems in each country. Belgium only has approximately 160 km^2^ of surface water according to the Food and Agriculture Organization database (Food and Agriculture Organization, 2019a, 2019b), which is only one‐third of that of Portugal (610 km^2^). The released FE shells were therefore much more diluted in Portugal than in Belgium, resulting in lower PEC values.

The potential hazardous effects posed by FE shells were evaluated for freshwater, sediment, and soil; and no detrimental effects to the tested organisms were observed even at the highest tested concentration. Therefore, only HONECs could be obtained from the experiments. To our knowledge, no other study has evaluated the ecotoxicity of FE shells or the materials they are made of, but there are numbers of studies carried out to investigate the ecological effects of microplastics (Anbumani & Kakkar, 2018; Wang et al., 2020). Adam et al. (2019) showed with a meta‐analysis that with the currently available quantitative information on NOEC values, the type of microplastic has no statistically significant effect on the toxicity. However, the mean freshwater PNEC reported for microplastics with 0.08 µg/L (Adam et al., 2019) is much smaller than the HONEC of 2.67 mg/L for *R. subcapitata* reported in the present study for FE shells. In terms of sediment, no negative effect was found for *L. variegatus* at the highest concentration (40% plastic weight of sediment mixture; Redondo‐Hasselerharm et al., 2018), which was in line with the results of FE shells (HONEC = 5.35 mg/kg). The toxicological data for the soil compartment are also very limited because most studies reported no significant effect on terrestrial organisms at the highest tested concentration (Sarker et al., 2020). One study found that microplastic at a concentration of 28% plastic weight of sediment can significantly reduce the growth rate of *Lumbricus terrestris* (Huerta Lwanga et al., 2016), but this tested concentration was much higher than the HONEC for FE shells (9.1 mg/kg). Therefore, ecotoxicological experiments at higher concentrations are needed to obtain NOEC/EC20 values for FE shells that can then be used in a standard environmental risk assessment by calculating PNEC values (European Chemicals Agency, 2008).

The environmental risks in our study were only characterized by directly comparing the distribution of PECs with the HONECs. All PECs estimated by the PMFA model were lower than the HONECs, indicating that currently no risk exists. Because the actual PNEC values that should be derived from experimentally observed NOEC values will be higher than HONECs, the current PEC/HONEC ratios tend to overestimate the risk. In addition, the inclusion of environmental fate processes will decrease the FE concentrations in water because the present study used a worst‐case scenario with no sedimentation from the water column. Microplastics will sediment from the water column in a size‐dependent way, and retention within a flowing river was predicted to be almost 100% for particles >50 µm (Besseling et al., 2017). The FE shells are therefore within a range where sedimentation can be expected, and thus the actual PECs will be lower. The lower PEC values will even further lower the PEC/HONEC ratios for FE shells, which will increase the spread between exposure and hazard.

On a longer timescale, the assessment of the environmental risks posed by FEs is subject to several uncertainties. On the hazard side, as discussed, actual effects, if any, probably occur at much higher concentrations than indicated by the HONECs used in the present study. On the exposure side, FEs are listed as one type of intentionally added microplastics which are proposed to be restricted after a transition period of 5 or 8 years by the European Chemicals Agency (2020a). In addition, the fragrance industry is striving to replace traditional FEs with biodegradable materials in the future. Therefore, it can be expected that the release of FEs made of traditional thermoset polymers is going to decrease in the next decade. Nevertheless, the microplastic global pollution coming from thermoplastic polymers in the environment is still far from being addressed.

In summary, a full risk assessment cannot be performed for FE shells at the moment because PNEC values cannot be calculated due to limited data. Therefore, PECs estimated by a PMFA model were compared with HONECs. The results suggest that current environmental levels of FE shells pose no risk to living organisms in freshwater, soil, and sediment for selected countries. Future studies need to be carried out to better understand the levels at which ecotoxicological effects are actually observed and the fate process of FE shells in the environment.

## Supplemental Data

The Supplemental Data are available on the Wiley Online Library at https://doi.org/10.1002/etc.5168.

## Disclaimer

Firmenich is a leading producer of fragrance encapsulates.

## Supporting information

This article includes online‐only Supplemental Data.

Supporting information.

## Data Availability

Data, associated metadata, and calculation tools are available from the corresponding author (nowack@empa.ch).
