# Supplementary material for: Country‐Specific Environmental Risks of Fragrance Encapsulates Used in Laundry Care Products
Source: Environ Toxicol Chem. 2021 Sep 2;41(4):905–16. doi: 10.1002/etc.5168 (PMC9291008; doi:10.1002/etc.5168)
Supplement: Supplementary file 1 — Supporting information. [file ETC-41-905-s001.pdf]

Country-specific environmental risks of fragrance encapsulates used in laundry care products

Supporting information

Number of figures: 4

Number of tables: 20

Table S1. Estimated masses of FE shells consumed in 34 countries in year of 2019. Unit: tons/year.

Country	Lower boundary	Mean	Upper boundary
Austria	1.3	2	2.8
Belgium	2.5	3.9	5.3
Bulgaria	0.69	1.1	1.5
Croatia	1.1	1.8	2.4
Cyprus	0.2	0.31	0.42
Czechia	1.7	2.6	3.5
Denmark	0.36	0.56	0.76
Estonia	0.13	0.2	0.28
Finland	0.48	0.74	1
France	11	17	24
Germany	20	31	43
Greece	1.5	2.3	3.1
Hungary	2.9	4.4	6.1
Ireland	0.54	0.84	1.20
Italy	12	19	26
Japan	29	58	87
Latvia	0.12	0.18	0.25
Lithuania	0.19	0.29	0.4
Luxembourg	0.13	0.19	0.26
Malta	0.09	0.14	0.19
Mexico	33	66	100
Netherlands	2.7	4.1	5.6
Norway	0.53	0.82	1.1
Poland	5.8	8.9	12
Portugal	3.5	5.3	7.3
Romania	3	4.6	6.3
Slovakia	0.98	1.5	2.1
Slovenia	0.35	0.53	0.73
Spain	19	29	40
Sweden	1.1	1.7	2.3
Switzerland	0.82	1.3	1.7
United Kingdom	11	17	23
USA	49	99	148
Vietnam	23	47	70

Table S2. Estimated trends of consumed amount of FE shells release from 2010 to 2019. Unit: unitless.

Year	Global	Japan	Mexico	USA	Vietnam
2010	0.23	0	0.16	0.13	0.26
2011	0.26	0	0.33	0.29	0.52
2012	0.82	0.04	0.65	0.6	1.04
2013	0.83	0.09	1.02	0.47	2.26
2014	0.97	0.17	1.02	0.86	2.43
2015	0.78	0.4	0.8	0.72	1.58
2016	0.77	0.84	0.83	0.75	1.35
2017	0.91	0.86	0.92	1.27	1.16
2018	1	0.9	0.96	0.98	1.15
2019	**1**	**1**	**1**	**1**	**1**

Table S3. WWTP removal efficiency of microplastics.

Study	Location	Removal efficiency
(Akarsu et al. 2020)	Turkey	0.57
(Bayo et al. 2020a)	Spain	0.988, 0.9553
(Bayo et al. 2020b)	Spain	0.903
(Conley et al. 2019)	USA	0.976, 0.852, 0.855
(Edo et al. 2020)	Spain	0.937
(Gies et al. 2018)	Canada	0.983
(Liu et al. 2019)	China	0.644
(Magni et al. 2019)	Italy	0.65
(Mintenig et al. 2017)	Germany	0.97
(Murphy et al. 2016)	UK	0.984
(Park et al. 2020)	Korea	0.995, 0.987, 0.994, 0.9977, 0.9995, 0.9999, 0.999, 0.9995, 0.99995, 0.999, 0.99994, 0.9993, 0.9998, 0.9998, 0.99973, 0.9988, 0.995, 0.995, 0.998, 0.9994, 0.9997, 0.998, 0.999, 0.999, 0.998, 0.967, 0.998, 0.9997, 0.999, 0.9999, 0.997
(Wang et al. 2020)	China	0.44, 0.98, 0.81, 0.35, 0.56, 0.78, 0.6, 0.54, 0.38
(Xu et al. 2019)	China	0.9059, 0.918, 0.9183, 0.9715, 0.9612, 0.9654, 0.9576, 0.8917, 0.9656, 0.9602, 0.9651, 0.9516
(Talvitie and Heinonen 2014)	Russia	0.9563
(Magnusson and Norén 2014)	Swedish	0.9969
(Dris et al. 2015)	Paris	0.89
(Talvitie et al. 2015)	Finland	0.98
(Carr et al. 2016)	USA	0.999
(Leslie et al. 2017)	Netherlands	0.72
(Ziajahromi et al. 2017)	Australia	0.9
(Gundogdu, Cevik et al. 2018	Turkey	0.79, 0.73
(Lares et al. 2018)	Finland	0.898
(Lee and Kim 2018)	Korea	0.985
(Simon et al. 2018)	Denmark	0.993
(Wiśniowskaa et al. 2018)	Poland	0.97
(Long et al. 2019)	China	0.951, 0.97, 0.995

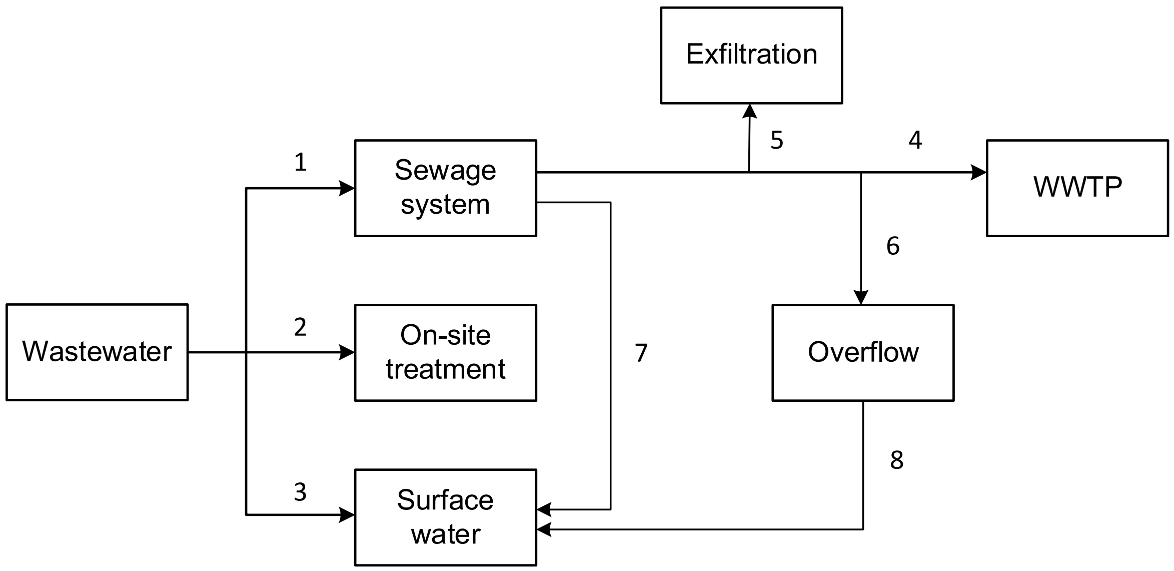

Figure S1. Pathways considered for wastewater management.

Table S4. Sources of transfer coefficients about wastewater management.

No of flow in Figure S1	Flow to	Source	Comments
1	Sewage system	European countries: (OECD 2019; Eurostat 2020b)<br>USA: (OECD 2019)<br>Mexico: (CNA 2018)<br>Japan: (OECD 2019)<br>Vietnam: (ARCOWA 2018)	The most recent data were extracted from the database
2	On-site treatment	Same as Rajkovic et al. (2020): the total amount equals to the fraction of wastewater not flowing to the sewer. Original data comes from (Cairns-Smith et al. 2014)	Including septic tanks, pit-dry and flush/pour flush pit (Rajkovic et al. 2020)<br>The value equals to (1-wastewater to sewer)*11/14
3	Surface water (from wastewater)		Including open defecation (Rajkovic et al. 2020)<br>The value equals to (1-wastewater to sewer)*3/14
4	WWTP	European countries: (OECD 2019; Eurostat 2020b)<br>USA: (OECD 2019)<br>Mexico: (CNA 2018)<br>Japan: (OECD 2019)<br>Vietnam: (ARCOWA 2018)	The most recent data were extracted from the database
5	Exfiltration	(Rutsch et al. 2006; Rajkovic et al. 2020)	Wastewater which is exfiltrated to the subsurface
6	Overflow	(Rajkovic et al. 2020)	
7	Surface water (from sewer)	Estimated from fraction of wastewater collected by sewer but not going to WWTP	
8	Surface water (from overflow)	Assuming 100% of overflow going to surface water for the countries	

Table S5. Sewage connection rate and WWTP treatment rate collected for each country.

Country	Source	Year	Sewer connection rate	WWTP treatment rate
Austria	OECD	2018	100%	96%
Belgium	Eurostat, OECD	2017	88%	83%
Bulgaria	Eurostat	2017	76%	63%
Croatia	Eurostat	2017	55%	53%
Cyprus	EEA, Eurostat	2014	42%	30%
Czechia	OECD	2018	82%	82%
Denmark	OECD	2018	100%	92%
Estonia	Eurostat, OECD	2017	83%	83%
Finland	OECD	2018	85%	85%
France	OECD	2018	81%	81%
Germany	Eurostat, OECD	2016	99%	97%
Greece	Eurostat, OECD	2016	93%	93%
Hungary	OECD	2018	80%	80%
Ireland	Eurostat, OECD	2017	64%	63%
Italy	EEA, Eurostat, OECD	2015, 2009	94%	63%
Japan	OECD	2017	79%	79%
Latvia	OECD	2018	80%	80%
Lithuania	OECD	2018	79%	76%
Luxembourg	Eurostat, OECD	2017	100%	99%
Malta	Eurostat	2017	99%	99%
Mexico	(CNA 2018)	-	91%	64%
Netherlands	OECD	2018	100%	100%
Norway	OECD	2018	87%	85%
Poland	OECD	2018	74%	74%
Portugal	Eurostat, OECD	2017	89%	88%
Romania	Eurostat	2017	51%	50%
Slovakia	Eurostat	2017	68%	67%
Slovenia	OECD	2018	72%	68%
Spain	OECD	2016	89%	88%
Sweden	Eurostat, OECD	2017	87%	87%
Switzerland	EEA, Eurostat, OECD	2015, 2013	98%	98%
United Kingdom	OECD, EEA	2015, 2010	100%	100%
USA	OECD	2012	76%	76%
Vietnam*	(ARCOWA 2018)	-	23%	13%

* For the urban area of Vietnam, the sewage connection rate was 60% and 21% of wastewater was reported to be treated by (ARCOWA 2018). Assumption was made for the rural area that 0% of wastewater was collected and treated. Then the final value was weighted by the percentage of urban and rural population (Worldbank 2018).

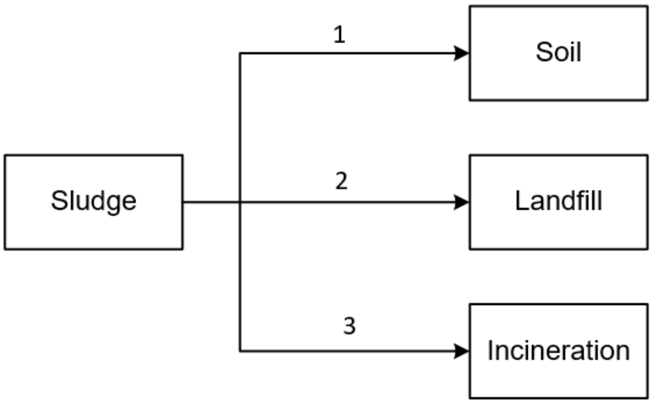

Figure S2. Pathways considered for sludge management.

Table S6. Source of transfer coefficients regarding sludge disposal.

No of flow in Figure S2	Flow to	Source	Comment
1	Soil	European countries: (OECD 2017; Eurostat 2020c)<br>USA: (Mahapatra et al. 2015)<br>Mexico: (CNA 2018)<br>Japan: (MLIT 2018)<br>Vietnam: (ARCOWA 2018)	The most recently available data were extracted from the database
2	Landfill		
3	Incineration		

Table S7. Data collected for sludge disposal.

Country	Source	Year	Agriculture	Landfill	Incineration
Austria	Eurostat	2016	43%	0%	57%
Belgium	Eurostat	2010	13%	0%	87%
Bulgaria	Eurostat	2017	79%	21%	0%
Croatia	Eurostat	2017	36%	64%	0%
Cyprus	Eurostat	2016	73%	0%	27%
Czechia	Eurostat	2013	90%	7.3%	2.4%
Denmark	Eurostat	2010	68%	1.3%	31%
Estonia	Eurostat	2009	83%	17%	0%
Finland	OECD	2010	98%	2.1%	0%
France	Eurostat	2017	79%	1.7%	19%
Germany	Eurostat	2016	35%	0%	65%
Greece	Eurostat	2014	29%	36%	35%
Hungary	Eurostat	2017	72%	0.55%	28%
Ireland	Eurostat	2017	100%	0%	0%
Italy	Eurostat	2010	39%	57%	4.5%
Japan	(MLIT 2018)	2018	15%	24%	61%
Latvia	Eurostat	2017	99%	0%	1.1%
Lithuania	Eurostat	2017	92%	7.9%	0%
Luxembourg	Eurostat	2017	81%	0%	19%
Malta	Eurostat	2017	0%	100%	0%
Mexico	(CNA 2018)	2018	40%	60%	0%
Netherlands	Eurostat	2016	1.3%	0%	98%
Norway	Eurostat	2017	86%	14%	0%
Poland	Eurostat	2017	53%	6.0%	42%
Portugal	Eurostat	2016	73%	27%	0%
Romania	Eurostat	2017	18%	82%	0%
Slovakia	Eurostat	2017	55%	18%	27%
Slovenia	Eurostat	2017	3.1%	2.3%	95%
Spain	Eurostat	2012	80%	16%	4.2%
Sweden	Eurostat	2016	94%	2.3%	3.2%
Switzerland	Eurostat	2013	0%	0%	100%
United Kingdom	Eurostat	2012	78%	0%	21%
USA	(Mahapatra et al. 2015)	-	54%	31%	15%
Vietnam*	(ARCOWA 2018)	-	20%	80%	0%

*According to the report, most of the sludge in Vietnam was dumped in landfill (ARCOWA 2018). Therefore, an assumption was made that 80% of the sludge flowed to landfill, while 20% was applied on agricultural soil.

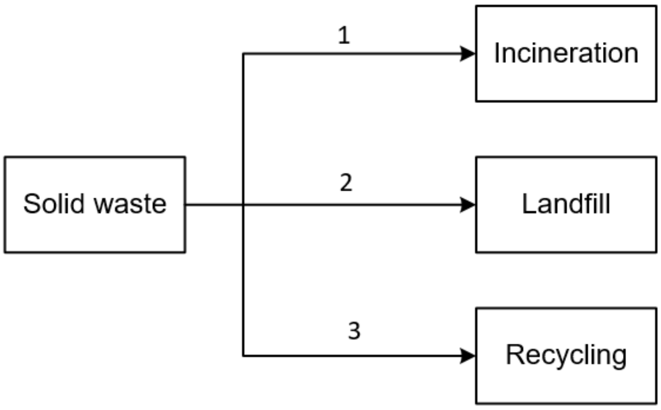

Figure S3. Pathways considered for solid waste management

Table S8. Source of transfer coefficients regarding solid waste management.

No of flow in Figure S3	Flow to	Source	Comment
1	Incineration	European countries: (Eurostat 2020a; OECD 2020)<br>USA, Mexico and Japan: (OECD 2020)<br>Vietnam: (Nguyen 2006)	The most recently available data were extracted from the database
2	Landfill		
3	Recycling		

Table S9. Data collected for solid waste management.

Country	Source	Year	Landfill	Incineration	Recycling
Austria	Eurostat, OECD	2018	3%	58%	38%
Belgium	Eurostat, OECD	2018	1.2%	55%	44%
Bulgaria	Eurostat	2018	62%	7.4%	30%
Croatia	Eurostat	2018	75%	0.10%	25%
Cyprus	Eurostat	2017	84%	0%	16%
Czechia	Eurostat	2018	53%	18%	29%
Denmark	Eurostat, OECD	2018	1.2%	60%	38%
Estonia	Eurostat, OECD	2018	25%	47%	28%
Finland	Eurostat, OECD	2018	0.80%	66%	34%
France	Eurostat, OECD	2018	26%	43%	31%
Germany	OECD	2018	0.30%	39%	61%
Greece	OECD	2017	84%	1.1%	15%
Hungary	Eurostat, OECD	2018	54%	15%	32%
Ireland	OECD	2017	26%	37%	37%
Italy	Eurostat, OECD	2018	31%	28%	42%
Japan	OECD	2017	1.0%	79%	20%
Latvia	Eurostat, OECD	2018	74%	2.4%	24%
Lithuania	Eurostat, OECD	2018	40%	20%	40%
Luxembourg	Eurostat, OECD	2018	7.9%	56%	36%
Malta	Eurostat	2018	93%	0%	7.0%
Mexico	OECD	2012	93%	0%	7.0%
Netherlands	Eurostat, OECD	2018	2.0%	60%	38%
Norway	Eurostat, OECD	2018	3.7%	60%	36%
Poland	Eurostat, OECD	2018	45%	26%	29%
Portugal	Eurostat, OECD	2018	62%	23%	16%
Romania	Eurostat	2018	86%	5.3%	8.9%
Slovakia	Eurostat	2018	61%	9.2%	30%
Slovenia	Eurostat, OECD	2018	15%	17%	68%
Spain	Eurostat, OECD	2018	62%	16%	22%
Sweden	Eurostat, OECD	2018	0.80%	64%	36%
Switzerland	Eurostat, OECD	2018	0%	61%	39%
United Kingdom	Eurostat, OECD	2018	18%	48%	34%
USA	OECD	2017	57%	14%	29%
Vietnam	(Nguyen 2006)	NA	100%	0%	0%

Table S10. Area of surface water. Two databases from FAO, land use and land cover databases, were considered (FAO 2019a, 2019b). Unit: 1000 ha.

Country	Land use	Land cover		Average*
		MODIS	CCI_LC	
Austria	136	25	71	92
Belgium	25	8.6	16	19
Bulgaria	244	64	125	169
Croatia	63	41	85	63
Cyprus	1.0	1.5	7.0	2.6
Czechia	166	5.1	60	99
Denmark	93	214	264	166
Estonia	187	202	223	200
Finland	3453	2001	3331	3060
France	153	222	430	239
Germany	788	213	447	559
Greece	306	224	342	295
Hungary	177	83	136	143
Ireland	139	120	197	149
Italy	720	275	402	529
Japan	1341	619	907	1052
Latvia	246	63	123	170
Lithuania	265	33	121	171
Luxembourg	16	0	0.70	8.2
Malta	NA	0.11	0.81	0.46
Mexico	2043	1187	1585	1715
Netherlands	372	78	120	236
Norway	2010	1357	2211	1897
Poland	651	137	454	473
Portugal	62	27	94	61
Romania	832	188	289	535
Slovakia	95	7.3	32	57
Slovenia	13	0.56	4.2	7.8
Spain	639	128	430	459
Sweden	4012	2145	3854	3506
Switzerland	177	103	142	150
United Kingdom	168	232	453	255
USA	68592	9210	18402	41199
Vietnam	2116	333	731	1324

* The average was calculated by giving a 50% weight to the values from land use and land cover databases.

Table S11. Volume of sludge. Unit: thousand tons.

Country	Source	Year	Total	Applied on agricultural soil
Austria	(Eurostat 2020c)	2016	238	102
Belgium	(Eurostat 2020c)	2010	131	17
Bulgaria	(Eurostat 2020c)	2017	45	36
Croatia	(Eurostat 2020c)	2017	3.4	1.2
Cyprus	(Eurostat 2020c)	2016	7.4	5.4
Czechia	(Eurostat 2020c)	2013	260	234
Denmark	(Eurostat 2020c)	2010	115	78
Estonia	(Eurostat 2020c)	2009	22	18
Finland	(OECD 2017)	2010	143	140
France	(Eurostat 2020c)	2017	809	639
Germany	(Eurostat 2020c)	2016	1773	621
Greece	(Eurostat 2020c)	2014	119	35
Hungary	(Eurostat 2020c)	2017	232	167
Ireland	(Eurostat 2020c)	2017	59	59
Italy	(Eurostat 2020c)	2010	954	372
Japan	(MLIT 2018)	2018	2279	342
Latvia	(Eurostat 2020c)	2017	25	25
Lithuania	(Eurostat 2020c)	2017	41	38
Luxembourg	(Eurostat 2020c)	2017	8.6	7.0
Malta	(Eurostat 2020c)	2017	10	0.0
Mexico	(CNA 2018)	2018	110	44
Netherlands	(Eurostat 2020c)	2016	325	4.2
Norway	(Eurostat 2020c)	2017	121	104
Poland	(Eurostat 2020c)	2017	585	310
Portugal	(Eurostat 2020c)	2016	119	87
Romania	(Eurostat 2020c)	2017	283	51
Slovakia	(Eurostat 2020c)	2017	55	30
Slovenia	(Eurostat 2020c)	2017	37	1.1
Spain	(Eurostat 2020c)	2012	1083	866
Sweden	(Eurostat 2020c)	2016	191	180
Switzerland	(Eurostat 2020c)	2013	195	0.0
United Kingdom	(Eurostat 2020c)	2012	1078	841
USA	(Mahapatra et al. 2015)	-	6514	3518
Vietnam*	(ARCOWA 2018), estimated	-	28	5.6

*The sludge volume for Vietnam was estimated from the total volume of wastewater treated per day (750 000 m3) according to the data provided by (ARCOWA 2018). Then the calculation was done via the website https://www.lenntech.com/wwtp/calculate-daily-sludge-production.htm and the typical parameter was taken. The annual sludge volume was estimated to be 27 700 t.

Calculation of PECs

$$PEC_{freshwater} = \frac{\sum_i Flow_{freshwater_i}}{Volume_{freshwater}} * \frac{Residence\ time}{365\ day}$$

Where:

$Flow_{freshwater_i}$: mass flows to the freshwater

$Volume_{freshwater}$: calculated by multiplying the area of the freshwater (Table S9) and the depth (3 m) as suggested by ECHA (2016)

$Residence\ time$: 40 days (ECHA 2016)

$$PEC_{sediment} = \frac{\sum_i Flow_{sediment_i}}{Volume_{sediment}}$$

Where:

$Flow_{sediment_i}$: mass flows to the sediment which equals to the flows to the freshwater

$Volume_{sediment}$: calculated by multiplying the area of the sediment which equals to freshwater area (Table S9), the depth (0.03 m) as suggested by ECHA (2016) and the density of dry sediment (260 kg/m^3) (Sun et al. 2014)

$$PEC_{Sludge-treated\ soil} = \frac{\sum_i Flow_{Sludge-treated\ soil_i}}{Mass_{Sludge-treated\ soil}}$$

$Flow_{Sludge-treated\ soil}$: mass flows to the sludge treated soil

$Mass_{Sludge-treated\ soil}$: calculated by multiplying the area, the depth and the density of the sludge-treated soil. The area was obtained by dividing the amount of sludge applied on soils (Table S10) by the application rate of 5000 kg/ha dry weight per year suggested by ECHA (2016). The depth of agricultural soil was 0.2 m (ECHA 2016) and the density was assumed to be 1500 kg/m^3 (Sun et al. 2014)

Table S12. Percentage of mass flows to different compartments.

Compartment	Europe	Japan	Mexico	USA	Vietnam
Sludge-treated soil	38%	9.5%	10%	32%	2.0%
Surface water	16%	13%	33%	13%	26%
Incineration	22%	41%	0%	10%	0%
Landfill	10%	15%	44%	21%	13%
Subsurface	14%	21%	12%	24%	59%

Table S13. PECs of FEs for the freshwater compartment.

Country	Unit	Q5	Mode	Mean	Q95
Austria	µg/L	0.0040	0.0057	0.012	0.037
Belgium	µg/L	0.056	0.078	0.13	0.34
Bulgaria	µg/L	0.0031	0.0042	0.0054	0.010
Croatia	µg/L	0.0094	0.014	0.017	0.035
Cyprus	µg/L	0.073	0.10	0.11	0.17
Czechia	µg/L	0.0045	0.0063	0.012	0.038
Denmark	µg/L	0.00090	0.0013	0.0022	0.0059
Estonia	µg/L	0.00018	0.00025	0.00048	0.0015
Finland	µg/L	0.000039	0.000056	0.00011	0.00036
France	µg/L	0.013	0.019	0.035	0.106
Germany	µg/L	0.0072	0.010	0.026	0.093
Greece	µg/L	0.00091	0.0013	0.0033	0.012
Hungary	µg/L	0.0057	0.0080	0.015	0.045
Ireland	µg/L	0.0017	0.0024	0.0033	0.0076
Italy	µg/L	0.030	0.043	0.049	0.078
Japan	µg/L	0.0095	0.014	0.026	0.078
Latvia	µg/L	0.00020	0.00028	0.00051	0.0015
Lithuania	µg/L	0.00045	0.00062	0.00097	0.0025
Luxembourg	µg/L	0.0028	0.0040	0.011	0.039
Malta	µg/L	0.027	0.039	0.12	0.49
Mexico	µg/L	0.026	0.040	0.047	0.080
Netherlands	µg/L	0.0017	0.0024	0.0074	0.028
Norway	µg/L	0.000086	0.00012	0.00022	0.00067
Poland	µg/L	0.0040	0.0057	0.0094	0.026
Portugal	µg/L	0.013	0.019	0.04	0.13
Romania	µg/L	0.0029	0.0041	0.0053	0.011
Slovakia	µg/L	0.0066	0.0095	0.014	0.035
Slovenia	µg/L	0.023	0.031	0.044	0.10
Spain	µg/L	0.011	0.015	0.031	0.099
Sweden	µg/L	0.000074	0.00010	0.00022	0.00073
Switzerland	µg/L	0.00087	0.0012	0.0036	0.014
United Kingdom	µg/L	0.0055	0.0080	0.027	0.11
USA	µg/L	0.00045	0.00069	0.001	0.0034
Vietnam	µg/L	0.020	0.032	0.034	0.050

Table S14. PECs of FEs for the sediment compartment.

Country	Unit	Q5	Mode	Mean	Q95
Austria	µg/kg	110	150	310	970
Belgium	µg/kg	1600	2100	3400	8800
Bulgaria	µg/kg	89	120	140	270
Croatia	µg/kg	270	360	460	930
Cyprus	µg/kg	2100	2800	3000	4300
Czechia	µg/kg	130	170	320	980
Denmark	µg/kg	25	35	57	150
Estonia	µg/kg	5.0	6.6	13	39
Finland	µg/kg	1.1	1.5	3.0	9.4
France	µg/kg	380	500	930	2800
Germany	µg/kg	200	270	690	2400
Greece	µg/kg	26	34	87	310
Hungary	µg/kg	160	220	400	1200
Ireland	µg/kg	48	63	88	200
Italy	µg/kg	860	1100	1300	2000
Japan	µg/kg	160	220	390	1200
Latvia	µg/kg	5.6	7.5	14	40
Lithuania	µg/kg	13	17	26	66
Luxembourg	µg/kg	78	110	280	1000
Malta	µg/kg	770	1000	3300	12000
Mexico	µg/kg	820	1100	1300	2100
Netherlands	µg/kg	47	65	200	740
Norway	µg/kg	2.5	3.1	5.9	17
Poland	µg/kg	120	150	250	690
Portugal	µg/kg	380	490	1100	3400
Romania	µg/kg	83	110	140	280
Slovakia	µg/kg	190	250	370	930
Slovenia	µg/kg	660	840	1200	2700
Spain	µg/kg	300	390	810	2500
Sweden	µg/kg	2.1	2.7	5.8	19
Switzerland	µg/kg	24	33	95	350
United Kingdom	µg/kg	150	220	720	2800
USA	µg/kg	13	17	29	82
Vietnam	µg/kg	1100	1400	1500	2100

Table S15. PECs of FEs for the sludge-treated soil compartment.

Country	Unit	Q5	Mode	Mean	Q95
Austria	µg/kg	45	85	82	110
Belgium	µg/kg	130	250	260	410
Bulgaria	µg/kg	83	160	150	190
Croatia	µg/kg	1400	2700	2700	4100
Cyprus	µg/kg	64	120	120	170
Czechia	µg/kg	47	86	81	99
Denmark	µg/kg	25	47	44	57
Estonia	µg/kg	45	81	76	95
Finland	µg/kg	26	48	44	54
France	µg/kg	100	180	170	220
Germany	µg/kg	95	180	180	250
Greece	µg/kg	95	180	180	250
Hungary	µg/kg	87	160	150	190
Ireland	µg/kg	50	94	88	110
Italy	µg/kg	66	130	120	180
Japan	µg/kg	58	110	110	170
Latvia	µg/kg	33	61	57	70
Lithuania	µg/kg	31	57	53	66
Luxembourg	µg/kg	130	240	220	270
Malta	µg/kg	0	0	0	0
Mexico	µg/kg	970	1900	1900	3100
Netherlands	µg/kg	63	120	130	220
Norway	µg/kg	33	61	57	71
Poland	µg/kg	61	120	110	150
Portugal	µg/kg	220	410	390	490
Romania	µg/kg	40	73	81	130
Slovakia	µg/kg	100	190	180	240
Slovenia	µg/kg	49	94	100	170
Spain	µg/kg	140	250	230	290
Sweden	µg/kg	45	83	77	93
Switzerland	µg/kg	0	0	0	0
United Kingdom	µg/kg	93	170	160	200
USA	µg/kg	58	110	100	140
Vietnam	µg/kg	1560	3000	3600	6200

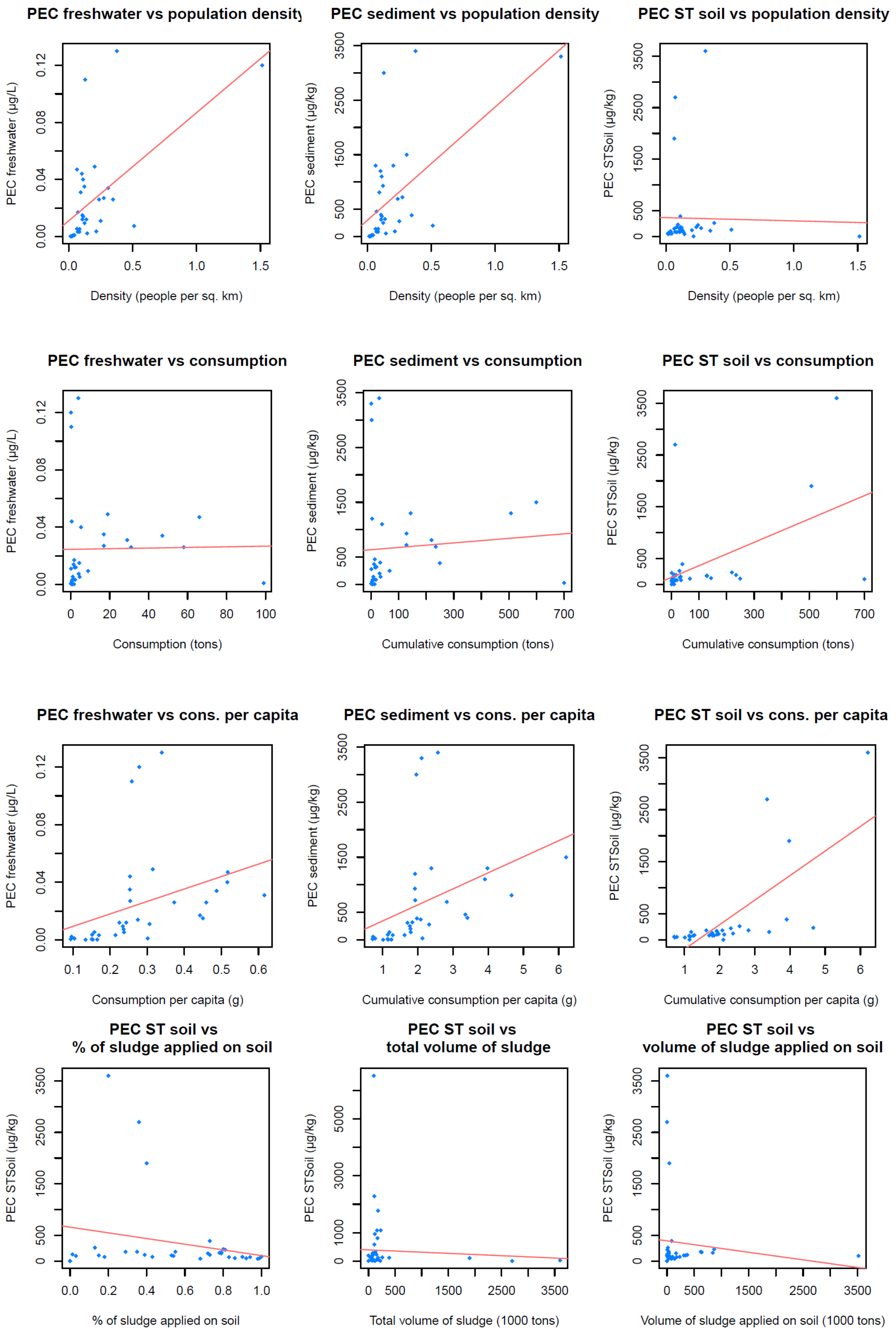
PEC freshwater vs population density
PEC freshwater (µg/L)
Density (people per sq. km)
PEC sediment vs population density
PEC sediment (µg/kg)
Density (people per sq. km)
PEC ST soil vs population density
PEC STSoil (µg/kg)
Density (people per sq. km)
PEC freshwater vs consumption
PEC freshwater (µg/L)
Consumption (tons)
PEC sediment vs consumption
PEC sediment (µg/kg)
Cumulative consumption (tons)
PEC ST soil vs consumption
PEC STSoil (µg/kg)
Cumulative consumption (tons)
PEC freshwater vs cons. per capita
PEC freshwater (µg/L)
Consumption per capita (g)
PEC sediment vs cons. per capita
PEC sediment (µg/kg)
Cumulative consumption per capita (g)
PEC ST soil vs cons. per capita
PEC STSoil (µg/kg)
Cumulative consumption per capita (g)
PEC ST soil vs
% of sludge applied on soil
PEC STSoil (µg/kg)
% of sludge applied on soil
PEC ST soil vs
total volume of sludge
PEC STSoil (µg/kg)
Total volume of sludge (1000 tons)
PEC ST soil vs
volume of sludge applied on soil
PEC STSoil (µg/kg)
Volume of sludge applied on soil (1000 tons)

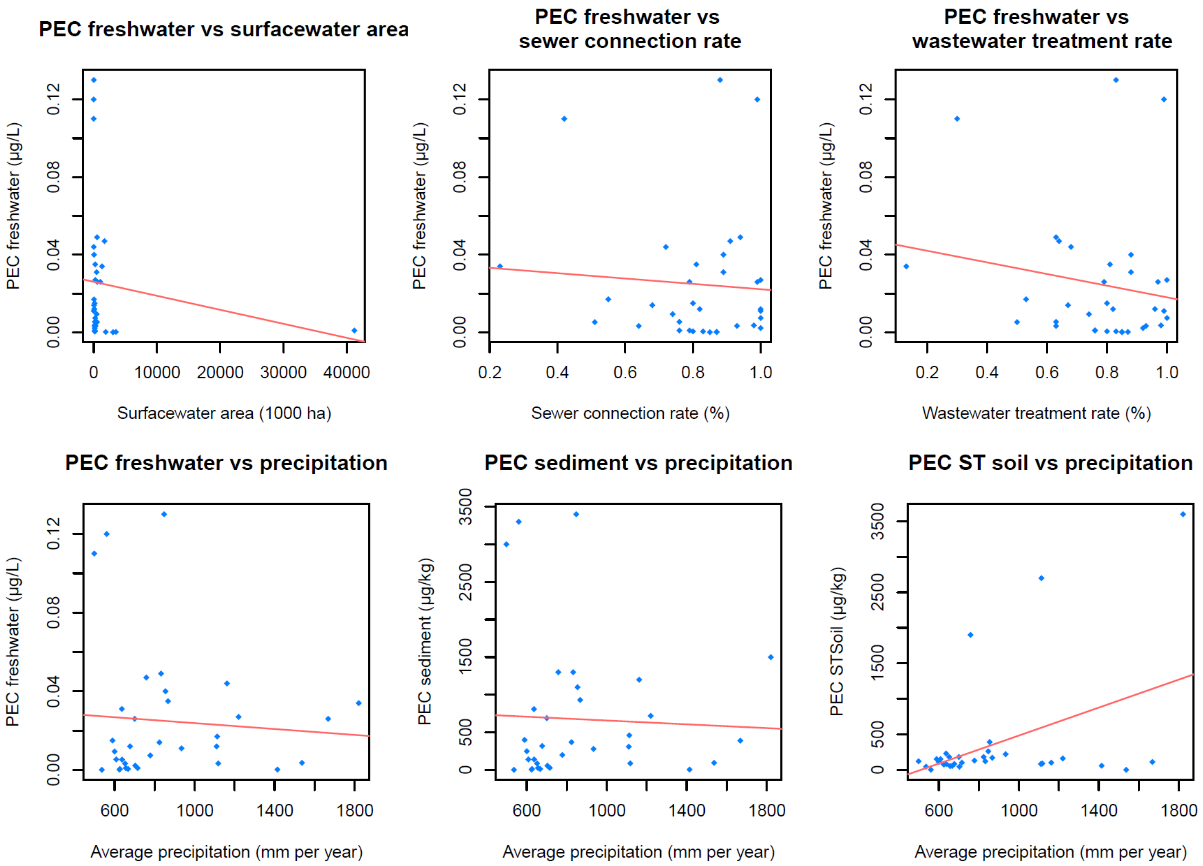

Figure S4. Correlation plots of PEC in water, sediment and sludge-treated (ST) soil vs. population density, consumption, consumption per capita, percentage of sludge applied on soil, total sludge volume, volume of sludge applied on soil, area of surface water, sewer connection rate, WWTP treatment rate, and precipitation.

Table S16. Statistical analysis of the correlations shown in Figure S4.

Correlation	Coefficients	Estimate	Pr(>|t|)	Signif.	R^2
PEC freshwater = a*Population density + b	b	0.011	0.065	.	0.33
	a	0.076	0.000	***	
PEC sediment = a*Population density + b	b	297.300	0.068	.	0.33
	a	2077.500	0.000	***	
PEC ST soil = a*Population density + b	b	364.370	0.036	*	-0.03
	a	-62.940	0.906		
PEC freshwater = a*Consumption + b	b	0.025	0.001	***	-0.03
	a	0.000	0.933		
PEC sediment = a*Cumulative consumption + b	b	634.405	0.002	**	-0.02
	a	0.414	0.656		
PEC ST soil = a*Cumulative consumption + b	b	134.230	0.327		0.23
	a	2.259	0.002	**	
PEC freshwater = a*Consumption per capita + b	b	0.001	0.955		0.09
	a	0.086	0.046	*	
PEC sediment = a*Cumulative consumption per capita + b	b	52.460	0.866		0.12
	a	291.300	0.028	*	
PEC ST soil = a*Cumulative consumption per capita + b	b	-656.040	0.002	**	0.50
	a	472.500	0.000	***	
PEC ST soil = a*% of sludge applied on soil+ b	b	658.800	0.018	*	0.02
	a	-553.300	0.188		
PEC ST soil = a*Total volume of sludge + b	b	396.243	0.012	*	-0.02
	a	-0.082	0.487		
PEC ST soil = a*Volume of sludge applied on soil + b	b	391.859	0.012	*	-0.02
	a	-0.147	0.510		
PEC freshwater = a*Surfacewater area + b	b	0.026	0.000	***	-0.01
	a	0.000	0.395		
PEC freshwater = a*Sewer connection rate + b	b	0.036	0.202		-0.03
	a	-0.014	0.681		
PEC freshwater = a*WWTP treatment rate + b	b	0.048	0.051	.	0.00
	a	-0.030	0.320		
PEC freshwater = a*Precipitation + b	b	0.031	0.065	.	-0.03
	a	0.000	0.674		
PEC sediment = a*Precipitation + b	b	784.443	0.090	.	-0.03
	a	-0.127	0.795		
PEC ST soil = a*Precipitation + b	b	-503.203	0.156		0.15
	a	0.985	0.013	*	

Signif. codes: 0 ‘***’ 0.001 ‘**’ 0.01 ‘*’ 0.05 ‘.’ 0.1 ‘ ’ 1

Table S17. Overview of ecotoxicological tests.

Compartment	Guideline	FE shells suspension	Species	Endpoint	Duration	Range (mg shells /L water or kg soil)	Effect
Fresh water	OECD 201	Commercial	*Raphidocelis subcapitata*	Growth	72 h	0.00535 to 0.0535	A 23.8% yield inhibition was observed at the lowest concentration
Fresh water	OECD 201	Purified	*Raphidocelis subcapitata*	Growth	72 h	0.0535 to 2.67	No effect the highest concentration
Fresh water	OECD 202	Commercial	*Daphnia magna*	immobilisation	72 h	0.00535 to 0.0535	No effect the highest concentration
Fresh water	OECD 236	Commercial	*Danio rerio*	survival or growth	96 h	0.00535 to 0.0535	No effect the highest concentration
Sediment	OECD 225	Commercial	*Lumbriculus variegatus*	mortality and weight	28 d	0.535 to 5.35	No effect the highest concentration
Soil	OECD 217	Commercial	Soil microorganisms	carbon transformation	28 d	0.535 to 5.35	No effect the highest concentration
Soil	ISO 15685	Commercial	Soil microorganisms	nitrogen transformation	28 d	0.535 to 5.35	No effect the highest concentration
Soil	OECD 208	Commercial	*Avena sativa*	growth	14 d	0.535 to 5.35	The control didn't meet the criteria
Soil	OECD 208	Purified	*Avena sativa*	emergence, survival and growth	14 d	1.09 to 9.1	No effect the highest concentration
Soil	OECD 208	Commercial	*Zea mays*	emergence, post-ermegence survival, growth	14 d	0.535 to 5.35	No effect the highest concentration
Soil	OECD 208	Commercial	*Phaseolus aureus*	shoot fresh weight	14 d	0.535 to 5.35	An effect on shoot fresh weight at the highest concentration and a NOEC value of 2.675 mg/kg
Soil	OECD 208	Purified	*Phaseolus aureus*	Emergence rate, post-emergence survival and biomass	14 d	1.09 to 9.1	No adverse effect at any concentration and a NOEC value > 9.1 mg/kg
Soil	OECD 208	Commercial	*Brassica rapa*	Emergence rate, post-emergence survival and biomass	14 d	0.535 to 5.35	No effect the highest concentration
Soil	OECD 222	Commercial	*Eisenia andrei*	reproduction	56 d	0.535 to 5.35	An effect on the reproduction at the mid and highest concentration and a NOEC value of 0.535 mg/kg
Soil	OECD 222	Purified	*Eisenia andrei*	reproduction, survival, weight	56 d	1.09 to 9.1	No adverse effect at any concentration and a NOEC value > 9.1 mg/kg

Table S18. A summary of the PEC/HONEC ratios for freshwater.

Country	Q5	Median	Mean	Q95
Austria	1.5E-06	2.8E-06	4.3E-06	1.4E-05
Belgium	2.1E-05	3.5E-05	4.7E-05	1.3E-04
Bulgaria	1.2E-06	1.8E-06	2.0E-06	3.9E-06
Croatia	3.5E-06	5.6E-06	6.5E-06	1.3E-05
Cyprus	2.7E-05	4.1E-05	4.2E-05	6.3E-05
Czechia	1.7E-06	3.0E-06	4.6E-06	1.4E-05
Denmark	3.4E-07	6.0E-07	8.2E-07	2.2E-06
Estonia	6.6E-08	1.2E-07	1.8E-07	5.6E-07
Finland	1.5E-08	2.6E-08	4.2E-08	1.3E-07
France	5.0E-06	8.7E-06	1.3E-05	4.0E-05
Germany	2.7E-06	5.4E-06	9.7E-06	3.5E-05
Greece	3.4E-07	6.7E-07	1.2E-06	4.4E-06
Hungary	2.1E-06	3.7E-06	5.5E-06	1.7E-05
Ireland	6.3E-07	1.0E-06	1.2E-06	2.9E-06
Italy	1.1E-05	1.7E-05	1.8E-05	2.9E-05
Japan	3.5E-06	6.7E-06	9.8E-06	2.9E-05
Latvia	7.4E-08	1.3E-07	1.9E-07	5.7E-07
Lithuania	1.7E-07	2.7E-07	3.6E-07	9.5E-07
Luxembourg	1.0E-06	2.2E-06	4.0E-06	1.4E-05
Malta	1.0E-05	2.3E-05	4.7E-05	1.8E-04
Mexico	9.7E-06	1.6E-05	1.8E-05	3.0E-05
Netherlands	6.3E-07	1.4E-06	2.8E-06	1.1E-05
Norway	3.2E-08	5.6E-08	8.3E-08	2.5E-07
Poland	1.5E-06	2.6E-06	3.5E-06	9.8E-06
Portugal	5.0E-06	9.0E-06	1.5E-05	4.9E-05
Romania	1.1E-06	1.7E-06	2.0E-06	4.0E-06
Slovakia	2.5E-06	4.1E-06	5.3E-06	1.3E-05
Slovenia	8.6E-06	1.4E-05	1.7E-05	3.8E-05
Spain	4.0E-06	7.2E-06	1.1E-05	3.7E-05
Sweden	2.8E-08	5.0E-08	8.2E-08	2.7E-07
Switzerland	3.3E-07	7.0E-07	1.4E-06	5.1E-06
United Kingdom	2.1E-06	4.9E-06	1.0E-05	4.1E-05
USA	1.7E-07	3.2E-07	4.4E-07	1.3E-06
Vietnam	7.5E-06	1.2E-05	1.3E-05	1.9E-05

Table S19. A summary of the PEC/HONEC ratios for sediment.

Country	Q5	Median	Mean	Q95
Austria	2.1E-02	3.7E-02	5.8E-02	1.8E-01
Belgium	3.0E-01	4.6E-01	6.3E-01	1.6E+00
Bulgaria	1.7E-02	2.4E-02	2.7E-02	5.1E-02
Croatia	5.0E-02	7.4E-02	8.6E-02	1.7E-01
Cyprus	4.0E-01	5.4E-01	5.6E-01	8.0E-01
Czechia	2.4E-02	3.9E-02	6.0E-02	1.8E-01
Denmark	4.7E-03	7.9E-03	1.1E-02	2.9E-02
Estonia	9.4E-04	1.5E-03	2.4E-03	7.3E-03
Finland	2.1E-04	3.5E-04	5.6E-04	1.8E-03
France	7.0E-02	1.1E-01	1.7E-01	5.2E-01
Germany	3.8E-02	7.2E-02	1.3E-01	4.5E-01
Greece	4.8E-03	8.9E-03	1.6E-02	5.7E-02
Hungary	3.0E-02	4.9E-02	7.3E-02	2.2E-01
Ireland	9.0E-03	1.3E-02	1.6E-02	3.7E-02
Italy	1.6E-01	2.3E-01	2.4E-01	3.8E-01
Japan	3.0E-02	5.0E-02	7.4E-02	2.2E-01
Latvia	1.0E-03	1.7E-03	2.5E-03	7.4E-03
Lithuania	2.4E-03	3.6E-03	4.8E-03	1.2E-02
Luxembourg	1.5E-02	2.9E-02	5.3E-02	1.9E-01
Malta	1.4E-01	3.1E-01	6.2E-01	2.3E+00
Mexico	1.5E-01	2.2E-01	2.4E-01	3.9E-01
Netherlands	8.8E-03	1.9E-02	3.7E-02	1.4E-01
Norway	4.6E-04	7.3E-04	1.1E-03	3.3E-03
Poland	2.1E-02	3.4E-02	4.7E-02	1.3E-01
Portugal	7.1E-02	1.2E-01	2.0E-01	6.4E-01
Romania	1.6E-02	2.3E-02	2.6E-02	5.2E-02
Slovakia	3.5E-02	5.4E-02	7.0E-02	1.7E-01
Slovenia	1.2E-01	1.8E-01	2.2E-01	5.0E-01
Spain	5.7E-02	9.4E-02	1.5E-01	4.8E-01
Sweden	3.9E-04	6.6E-04	1.1E-03	3.5E-03
Switzerland	4.5E-03	9.2E-03	1.8E-02	6.6E-02
United Kingdom	2.8E-02	6.5E-02	1.3E-01	5.2E-01
USA	2.4E-03	3.9E-03	5.5E-03	1.5E-02
Vietnam	2.0E-01	2.8E-01	2.8E-01	3.8E-01

Table S20. A summary of the PEC/HONEC ratios for sludge-treated soil.

Country	Q5	Median	Mean	Q95
Austria	4.9E-03	9.1E-03	9.0E-03	1.2E-02
Belgium	1.4E-02	2.8E-02	2.9E-02	4.5E-02
Bulgaria	9.2E-03	1.7E-02	1.6E-02	2.1E-02
Croatia	1.5E-01	3.0E-01	3.0E-01	4.5E-01
Cyprus	7.1E-03	1.3E-02	1.3E-02	1.9E-02
Czechia	5.2E-03	9.2E-03	8.9E-03	1.1E-02
Denmark	2.8E-03	5.0E-03	4.9E-03	6.2E-03
Estonia	4.9E-03	8.7E-03	8.4E-03	1.0E-02
Finland	2.9E-03	5.1E-03	4.9E-03	5.9E-03
France	1.1E-02	2.0E-02	1.9E-02	2.4E-02
Germany	1.0E-02	2.0E-02	2.0E-02	2.8E-02
Greece	1.0E-02	2.0E-02	1.9E-02	2.7E-02
Hungary	9.5E-03	1.7E-02	1.7E-02	2.1E-02
Ireland	5.5E-03	1.0E-02	9.7E-03	1.2E-02
Italy	7.2E-03	1.4E-02	1.4E-02	2.0E-02
Japan	6.4E-03	1.2E-02	1.3E-02	1.9E-02
Latvia	3.7E-03	6.5E-03	6.3E-03	7.7E-03
Lithuania	3.4E-03	6.1E-03	5.9E-03	7.3E-03
Luxembourg	1.4E-02	2.5E-02	2.4E-02	3.0E-02
Malta	0	0	0	0
Mexico	1.1E-01	2.1E-01	2.1E-01	3.4E-01
Netherlands	7.0E-03	1.4E-02	1.4E-02	2.4E-02
Norway	3.7E-03	6.6E-03	6.3E-03	7.8E-03
Poland	6.7E-03	1.2E-02	1.2E-02	1.7E-02
Portugal	2.5E-02	4.5E-02	4.3E-02	5.4E-02
Romania	4.3E-03	8.7E-03	8.9E-03	1.4E-02
Slovakia	1.1E-02	2.0E-02	2.0E-02	2.7E-02
Slovenia	5.4E-03	1.1E-02	1.1E-02	1.9E-02
Spain	1.5E-02	2.7E-02	2.6E-02	3.2E-02
Sweden	5.0E-03	8.8E-03	8.4E-03	1.0E-02
Switzerland	0	0	0	0
United Kingdom	1.0E-02	1.8E-02	1.7E-02	2.2E-02
USA	6.4E-03	1.2E-02	1.2E-02	1.6E-02
Vietnam	1.7E-01	3.7E-01	3.9E-01	6.8E-01

References

1. Akarsu C, Kumbur H, Gokdag K, Kideys AE, Sanchez-Vidal A. 2020. Microplastics composition and load from three wastewater treatment plants discharging into Mersin Bay, north eastern Mediterranean Sea. *Marine Pollution Bulletin* 150:110776.
2. ARCOWA. 2018. Wastewater management and resource recovery in vietnam: Current status and opportunities.
3. Bayo J, Lopez-Castellanos J, Olmos S. 2020a. Membrane bioreactor and rapid sand filtration for the removal of microplastics in an urban wastewater treatment plant. *Mar Pollut Bull* 156:111211.
4. Bayo J, Olmos S, Lopez-Castellanos J. 2020b. Microplastics in an urban wastewater treatment plant: The influence of physicochemical parameters and environmental factors. *Chemosphere* 238:124593.
5. Cairns-Smith S, Hill H, Nazarenko E. 2014. Urban Sanitation: Why a portfolio of solutions is needed, Working Paper.
6. Carr SA, Liu J, Tesoro AG. 2016. Transport and fate of microplastic particles in wastewater treatment plants. *Water Res* 91:174-182.
7. CNA. 2018. Estadisticas del Agua en México, Comisión Nacional del Agua.
8. Conley K, Clum A, Deepe J, Lane H, Beckingham B. 2019. Wastewater treatment plants as a source of microplastics to an urban estuary: Removal efficiencies and loading per capita over one year. *Water Research X* 3:100030.
9. Dris R, Gasperi J, Rocher V, Saad M, Renault N, Tassin B. 2015. Microplastic contamination in an urban area: a case study in Greater Paris. *Environmental Chemistry* 12:592-599.
10. ECHA. 2016. Guidance on information requirements and Chemical Safety Assessment
11. Chapter R.16: Environmental exposure assessment.
12. Edo C, Gonzalez-Pleiter M, Leganes F, Fernandez-Pinas F, Rosal R. 2020. Fate of microplastics in wastewater treatment plants and their environmental dispersion with effluent and sludge. *Environmental Pollution* 259:113837.
13. Eurostat. 2020a. Municipal waste by waste management operations. *https://appssoeurostateceuropaeu/nui/showdo?dataset=env_wasmun&lang=en (Data extracted 5 April 2020)*.
14. Eurostat. 2020b. Population connected to urban wastewater collecting and treatment systems, by treatment level. *https://eceuropaeu/eurostat/tgm/tabledo?tab=table&init=1&plugin=1&language=en&pcode=ten00020 (Data extracted 5 April 2020)*.
15. Eurostat. 2020c. Sewage sludge production and disposal. *https://appssoeurostateceuropaeu/nui/showdo?dataset=env_ww_spd&lang=en*.
16. FAO. 2019a. FAOSTAT Agri-Environmental Indicators, Land Cover. *http://wwwfaoorg/faostat/en/#data/LC (Data extracted 10 April 2020)*.
17. FAO. 2019b. FAOSTAT Inputs/Land Use domain. *http://wwwfaoorg/faostat/en/#data/RL (Data extracted 10 April 2020)*.
18. Gies EA, LeNoble JL, Noel M, Etemadifar A, Bishay F, Hall ER, Ross PS. 2018. Retention of microplastics in a major secondary wastewater treatment plant in Vancouver, Canada. *Mar Pollut Bull* 133:553-561.
19. Lares M, Ncibi MC, Sillanpaa M, Sillanpaa M. 2018. Occurrence, identification and removal of microplastic particles and fibers in conventional activated sludge process and advanced MBR technology. *Water Res* 133:236-246.
20. Lee H, Kim Y. 2018. Treatment characteristics of microplastics at biological sewage treatment facilities in Korea. *Marine Pollution Bulletin* 137:1-8.
21. Leslie HA, Brandsma SH, van Velzen MJ, Vethaak AD. 2017. Microplastics en route: Field measurements in the Dutch river delta and Amsterdam canals, wastewater treatment plants, North Sea sediments and biota. *Environ Int* 101:133-142.
22. Liu XN, Yuan WK, Di MX, Li Z, Wang J. 2019. Transfer and fate of microplastics during the conventional activated sludge process in one wastewater treatment plant of China. *Chem Eng J* 362:176-182.

23. Long Z, Pan Z, Wang W, Ren J, Yu X, Lin L, Lin H, Chen H, Jin X. 2019. Microplastic abundance, characteristics, and removal in wastewater treatment plants in a coastal city of China. *Water Res* 155:255-265.
24. Magni S, Binelli A, Pittura L, Avio CG, Della Torre C, Parenti CC, Gorbi S, Regoli F. 2019. The fate of microplastics in an Italian Wastewater Treatment Plant. *Sci Total Environ* 652:602-610.
25. Magnusson K, Norén F. 2014. Screening of microplastic particles in and down-stream a wastewater treatment plant.
26. Mahapatra I, Sun TY, Clark JR, Dobson PJ, Hungerbuehler K, Owen R, Nowack B, Lead J. 2015. Probabilistic modelling of prospective environmental concentrations of gold nanoparticles from medical applications as a basis for risk assessment. *J Nanobiotechnol* 13:93.
27. Mintenig SM, Int-Veen I, Loder MGJ, Primpke S, Gerdts G. 2017. Identification of microplastic in effluents of waste water treatment plants using focal plane array-based micro-Fourier-transform infrared imaging. *Water Res* 108:365-372.
28. MLIT. 2018. 資源・エネルギー循環の形成, Ministry of Land, Infrastructure, Transport and Tourism. *https://wwwmlitgojp/mizukokudo/sewerage/crd_sewerage_tk_000124html*.
29. Murphy F, Ewins C, Carbonnier F, Quinn B. 2016. Wastewater Treatment Works (WwTW) as a Source of Microplastics in the Aquatic Environment. *Environ Sci Technol* 50:5800-5808.
30. Nguyen T. 2006. Solid waste management in Vietnam-An industrial ecology study. Columbia University.
31. OECD. 2017. Water: Sewage sludge production and disposal (Edition 2017). *ISSN: 18169465 (online) https://doiorg/101787/env-data-en*.
32. OECD. 2019. Water: Wastewater treatment (Edition 2019). *https://wwwoecd-ilibraryorg/environment/data/oecd-environment-statistics/water-wastewater-treatment-edition-2019_2ba9fed5-en*.
33. OECD. 2020. Municipal waste, Generation and Treatment. *https://statsoecdorg/Indexaspx?DataSetCode=MUNW (Data extracted 5 April 2020)*.
34. Park HJ, Oh MJ, Kim PG, Kim G, Jeong DH, Ju BK, Lee WS, Chung HM, Kang HJ, Kwon JH. 2020. National Reconnaissance Survey of Microplastics in Municipal Wastewater Treatment Plants in Korea. *Environ Sci Technol* 54:1503-1512.
35. Rajkovic S, Bornhöft NA, van der Weijden R, Nowack B, Adam V. 2020. Dynamic probabilistic material flow analysis of engineered nanomaterials in European waste treatment systems. *Waste Management* 113:118-131.
36. Rutsch M, Rieckermann J, Krebs P. 2006. Quantification of sewer leakage: a review. *Water Science and Technology* 54:135-144.
37. Simon M, van Alst N, Vollertsen J. 2018. Quantification of microplastic mass and removal rates at wastewater treatment plants applying Focal Plane Array (FPA)-based Fourier Transform Infrared (FT-IR) imaging. *Water Res* 142:1-9.
38. Sun TY, Gottschalk F, Hungerbühler K, Nowack B. 2014. Comprehensive probabilistic modelling of environmental emissions of engineered nanomaterials. *Environmental pollution* 185:69-76.
39. Talvitie J, Heinonen M. 2014. Preliminary study on synthetic microfibers and particles at a municipal waste water treatment plant. *Balt Mar Environ Prot Comm HELCOM, Helsinki*:1-14.
40. Talvitie J, Heinonen M, Pääkkönen J-P, Vahtera E, Mikola A, Setälä O, Vahala R. 2015. Do wastewater treatment plants act as a potential point source of microplastics? Preliminary study in the coastal Gulf of Finland, Baltic Sea. *Water Science and Technology* 72:1495-1504.
41. Wang F, Wang B, Duan L, Zhang Y, Zhou Y, Sui Q, Xu D, Qu H, Yu G. 2020. Occurrence and distribution of microplastics in domestic, industrial, agricultural and aquacultural wastewater sources: A case study in Changzhou, China. *Water Res* 182:115956.
42. Wiśniowskaa E, Moraczewska-Majkutb K, Nocońb W. 2018. Efficiency of microplastics removal in selected wastewater treatment plants–preliminary studies. *Water Treat* 134:316-323.
43. Worldbank. 2018. Urban population. *United Nations Population Division World Urbanization Prospects: 2018 Revision https://dataworldbankorg/indicator/SPURBTOTLINZS?name_desc=true (Data extracted 10 April 2020)*.
44. Xu X, Jian Y, Xue Y, Hou Q, Wang L. 2019. Microplastics in the wastewater treatment plants (WWTPs): Occurrence and removal. *Chemosphere* 235:1089-1096.

45. Ziajahromi S, Neale PA, Rintoul L, Leusch FDL. 2017. Wastewater treatment plants as a pathway for microplastics: Development of a new approach to sample wastewater-based microplastics. *Water Res* 112:93-99.
